# Synthesis of Low Cost Titanium Silicalite-1 Zeolite for Highly Efficient Propylene Epoxidation

**DOI:** 10.3389/fchem.2021.682404

**Published:** 2021-06-07

**Authors:** Meng Liu, Zihan Huang, Wei Wei, Xiangyu Wang, Yiqiang Wen

**Affiliations:** ^1^ Henan Engineering Center of New Energy Battery Materials, Henan D&A Engineering Center of Advanced Battery Materials, College of Chemistry and Chemical Engineering, Shangqiu Normal University, Shangqiu, China; ^2^ Green Catalysis Center, and College of Chemistry, Zhengzhou University, Zhengzhou, China

**Keywords:** green synthesis, low-cost, small crystals, TS-1 zeolite, propylene epoxidation

## Abstract

Developing an effective and low-cost system to synthesize titanium silicalite-1 (TS-1) zeolite is desirable for a range of industrial applications. To date, the poor catalytic activity of the synthesized zeolite due to the low amount of framework titanium and large crystal size is the main obstacle limiting the widespread application of this material. Moreover, a large amount of wastewater is often produced by the existing synthesis process. Herein, a green and sustainable route for synthesizing small-crystal TS-1 with a high fraction of framework Ti was demonstrated via a seed-assisted method using a tetrapropylammonium bromide (TPABr)-ethanolamine hydrothermal system. The influence of the synthesis conditions on the physicochemical properties and catalytic activities of TS-1 was investigated. With the assistance of nanosized S-1 seeds, the incorporation of Ti into the framework of TS-1 was promoted, and the crystallization rate was effectively accelerated. After alkaline etching, the obtained hierarchical TS-1 had higher catalytic activity towards propylene epoxidation with an extremely high turnover frequency of 1,650 h^−1^. Furthermore, the mother liquid during the hydrothermal reaction could be reused for the next synthesis procedure. Consequently, utilization ratios of both ethanolamine and TPABr exceeding 95% were achieved by recycling the mother liquid. This low-cost approach for reducing wastewater could be easily scaled up to provide a promising synthesis method for the industrial production of TS-1 and other topological zeolites.

## Introduction

Since the titanium silicalite-1 (TS-1) zeolite reported by Taramasso et al. (1983), it has been widely used in many green oxidation reactions with hydrogen peroxide as oxidant owing to its unique catalytic performance in alcohol and alkane oxidation (Huybrechts et al., 1990; Fan et al., 2008; Liu et al., 2017), aromatic hydroxylation (Han et al., 2018), ketone ammoximation (Hu et al., 2018; Wang et al., 2018; Niu et al., 2019), oxidative desulfurization (Bai et al., 2018; Du et al., 2019) and alkene epoxidation (Feng et al., 2018; Harris et al., 2018; Zhang et al., 2018; Yuan et al., 2020). Generally, nanosized TS-1 zeolites (100–200 nm) are prepared using an organic silicon source (tetraethyl orthosilicalite), organometallic titanium source (tetraethyl orthotitanate), and a large amount of a template material (tetrapropylammonium hydroxide, TPAOH) (Carati et al., 1999). Although TS-1 synthesized using TPAOH as a template has been commercialized, the high price and thermal decomposition of TPAOH (Yaremov et al., 2007) lead to high preparation cost of TS-1 and the presence of tripropylamine in the synthetic mother liquid, respectively. Many efforts have been made to reduce the production cost of TS-1, including minimizing the usage of TPAOH (Deng et al., 2013; Huang et al., 2013). With the assistance of a non-ionic surfactant, the molar ratio of TPAOH/SiO_2_ was decreased to 0.08 (Karimi Haghighi and Nemati Kharat, 2021). Using a solid transformation method, the TPAOH/SiO_2_ molar ratio was further reduced to 0.05 (Zhang et al., 2015). Nevertheless, excessive template material is still required, which is difficult to recycle owing to its thermal decomposition (Zhai et al., 2006). Other alternative approaches are based on the use of inexpensive raw materials (Zhou and Wang, 2000; Wang et al., 2012; Xue et al., 2019). As a cheap template, tetrapropylammonium bromide (TPABr) has been widely used in the synthesis of TS-1 zeolite because of its good thermal stability and low price which is only about one third of that of TPAOH. For example, TS-1 has been prepared using TPABr as the template material (Müller and Steck, 1994), while TS-1 zeolite with a large crystal size of 6 μm was achieved using TPABr and n-butylamine as the template and base, respectively (Wang et al., 2002). Furthermore, alkoxide-free synthesis of TS-1 was achieved using amorphous silica fume and crystalline anatase-type titania nanoparticles as the silicon and titanium sources, respectively (Iwasaki et al., 2012). However, these TS-1 samples usually have large crystal sizes (>1 μm) and low fractions of framework titanium, which limit their catalytic activity because of poor diffusion properties and an insufficient number of active sites (Madsen et al., 1999; Zhang et al., 2017). Therefore, developing an effective strategy to synthesize TS-1 zeolite with high catalytic activity via a low-cost process is of great importance to enable its further application (Wang et al., 2012; Ye et al., 2015).

Additionally, given the global transition to industrial processes with reduced environmental impact, the green synthesis of TS-1 is also an urgent technical problem. A large amount of mother liquid containing some raw materials is produced during the synthesis of TS-1 (Xiong et al., 2016; Zhang et al., 2016). If the mother liquid is discharged directly as waste, it becomes a source of serious environmental pollution and reduces the utilization efficiency of the raw materials, leading to a high production cost. Hence, the reuse of the mother liquid is of environmental and economic significance.

In this study, a green and sustainable synthesis method for TS-1 with high catalytic activity was developed where the TS-1 crystals are nucleated from seeds in a TPABr-ethanolamine hydrothermal system. The synthesis conditions were systematically studied, including the Ti source, Si/Ti molar ratio [*n* (Si/Ti)], and crystallization time. Moreover, the recycling of the mother liquid was explored to increase the utilization ratios of the precursor materials and reduce the waste produced. The catalytic performance of the TS-1 sample synthesized using this method was evaluated using the hydrogen peroxide to propylene oxide (HPPO) conversion process.

## Materials and Methods

### Synthesis of Titanium Silicalite-1

The raw TS-1 powder was hydrothermally synthesized from the sol with a molar composition of SiO_2_: *n* TiO_2_: 0.1 TPABr: 0.5 NH_2_C_2_H_4_OH: 30 H_2_O (*n* = 0.018, 0.020, 0.022, 0.025, 0.029, 0.033). Typically, ethanolamine (EOA) and TPABr were mixed with deionized water, and fumed silica was added to the mixture under stirring (solution A). Under vigorous stirring, solution B of tetrabutyl titanate (TBOT) and isopropanol was added dropwise to solution A to form solution C. A suspension of nanosized S-1 zeolite seeds was added to solution C (Liu et al., 2016), and then the resulting sol was further stirred at 25°C for 24 h. Finally, the sol was crystallized at 175°C in a Teflon-lined autoclave under stirring for 6–72 h. The as-synthesized solid was centrifuged and washed with water and then dried at 120°C overnight, followed by calcination at 550°C for 6 h. The filtrate obtained in the centrifugation was reused in the subsequent synthesis. The method for determining the concentrations of EOA and TPABr in the filtrate is listed in Supplementary Material. To investigate the influence of the Ti source on the synthesis process, TBOT was replaced by Ti(SO_4_)_2_ or TiCl_3_. Supplementary Table S1 summarizes the detailed synthesis conditions of all samples.

Furthermore, a hierarchical TS-1 catalyst (denoted as HTS-1) was synthesized by alkaline modification of TS-1-35 (Liu et al., 2018). Nanosized TS-1 (denoted as NTS-1) was prepared as reported for comparison (Thangaraj et al., 1992).

### Mother Liquid Recycling Process

The mother liquid produced in the above process was kept and used in the subsequent synthesis of TS-1. The molar composition of added materials was SiO_2_: 0.029 TiO_2_: *x* TPABr: *y* NH_2_C_2_H_4_OH: 30 H_2_O (*x* = 0, 0.025, 0.050, 0.075, 0.100; *y* = 0, 0.125, 0.250, 0.375, 0.500), in which the H_2_O was completely replaced by mother liquid. EOA, TPABr and other traces of raw materials that may exist in mother liquid were not considered. The specific steps were consistent with “Synthesis of Titanium Silicalite-1” section. Then the obtained mother liquid in this batch was continued to be recycled in the next batch. The yield of TS-1 in each batch was calculated on the basis of the mass of the final solid TS-1 divided by the added mass of SiO_2_ and TiO_2_ in precursor sol.

### Propylene Epoxidation

**SCHEME 1:**
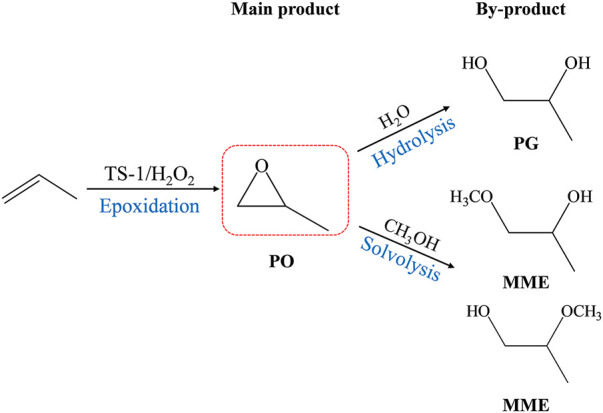
Reaction routes of TS-1/H2O2-catalyzed HPPO process.

The propylene epoxidation reaction was performed in a 200 ml stainless-steel reactor with the TS-1 samples used as the catalyst. The TS-1 raw powder (0.5 g), methanol (24 ml), and H_2_O_2_ (3 ml, 27.5 wt%) were added to the reactor, then the mixture was heated to 45°C under stirring. Finally, 0.6 MPa of propylene was charged into the system, and then the reaction was left to run for 1 h. In this reaction system (Scheme 1), the main product was propylene oxide (PO), and the main byproducts were propylene glycol (PG) and propylene glycol monomethyl ethers (MME).

A detailed analysis of the reaction results is given in Supplementary Material. The conversion of H_2_O_2_ (*X*_H2O2_), yield of propylene oxide (*Y*_PO_), selectivity to propylene oxide (*S*_PO_), and utilization of H_2_O_2_ (*U*_H2O2_) were calculated using Supplementary Equations S1–S4, respectively.

Turnover frequency (TOF) was calculated on the basis of the efficient conversion of H_2_O_2_ per hour divided by the amount of Ti species in the TS-1 zeolite. The detailed calculation was as follows:

$$\text{TOF}=n_{0}\left({\text{H}}_{2}{\text{O}}_{2}\right)\times X_{{\text{H}}_{2}{\text{O}}_{2}}\times U_{{\text{H}}_{2}{\text{O}}_{2}}/\left(n_{\mathrm{Ti}}\times T\right)$$

The *n*_0_(H_2_O_2_) represents the initial molar amounts of H_2_O_2_. The *n*_Ti_ represents the molar amounts of Ti of catalyst measured by inductively coupled plasma (ICP) spectroscopy. The *X*_H2O2_ and *U*_H2O2_ stand for the conversion of H_2_O_2_ and utilization of H_2_O_2_, respectively. The *T* represents the time on stream.

### Characterizations

The crystalline structures of samples were characterized by X-ray powder diffraction (XRD) pattern. The relative crystallinity (RC) was estimated by comparing the total integrated intensity of the characteristic peaks of each sample, and the intensity of sample TS-1-0.6EOA was set to 100% to normalize the patterns of all samples (Zhou et al., 2010). Fourier transform infrared (FT-IR) and Ultraviolet–visible (UV–Vis) spectroscopy were used to detect the coordination state of the Ti species, and the height of the adsorption peak was used to represent the peak intensity qualitatively. The amounts of SiO_2_ and TiO_2_ in the bulk and on the surface of obtained zeolites were measured via inductively coupled plasma (ICP) spectroscopy and X-ray photoelectron spectra (XPS), respectively. Nitrogen sorption measurements were used to analyze the textural properties of samples. The morphology and porous structure of TS-1 were analyzed via scanning electron microscope (SEM) and transmission electron microscope (TEM), respectively.

The details of the instruments and analysis methods are presented in the Supplementary Material.

## Results and Discussion

### Synthesis Conditions

#### Titanium Source

**FIGURE 1:**
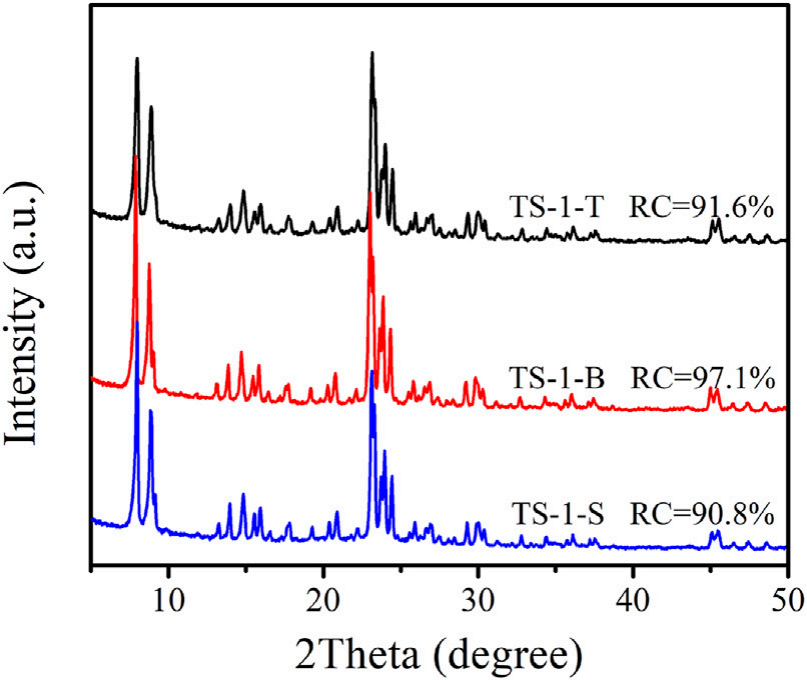
XRD diffractograms of samples with titanium source: TiCl_3_ (TS-1-T), TBOT (TS-1-B), and Ti(SO_4_)_2_ (TS-1-S).

The matching hydrolysis rates of titanium and silicon sources would be beneficial for increasing the content of Ti inserted into the framework. Therefore, three TS-1 samples were synthesized with TiCl_3_, TBOT, and Ti(SO_4_)_2_ as titanium sources, henceforth referred to as TS-1-T, TS-1-B, and TS-1-S, respectively. These samples were prepared with all other synthesis parameters [*n* (Si/Ti) = 50 in the precursor sol; crystallization time of 24 h]. Figure 1 shows the XRD diffractograms of these samples. Characteristic peaks at 2*θ* = 7.8°, 8.8°, 23.0°, 23.9°, and 24.4° were observed for all samples, which indicated their MFI topology. When TBOT was used as a Ti source, the obtained TS-1-B powder had a higher RC than the other two samples. The lower RC of TS-1-T and TS-1-S was attributed to the lower alkalinity of the hydrothermal systems when TiCl_3_ and Ti(SO_4_)_2_ were added compared to when TBOT was used; the pH values of these hydrothermal systems are listed in Supplementary Table S2, van der Pol and van Hooff, 1992). The SEM and TEM images of these samples are shown in Supplementary Figures S1,S2, respectively. It can be seen that sample TS-1-B was in the form of a rounded-boat morphology (Roeffaers et al., 2008), and the crystal size (1.09 × 0.55 × 0.22 μm) was smaller than that of the other two samples. The chemical compositions of the bulk of the three samples are listed in Supplementary Table S3. TS-1-B had a slightly higher Ti content than the other samples, which may be due to a larger number of Ti atoms inserted into the framework.

**FIGURE 2:**
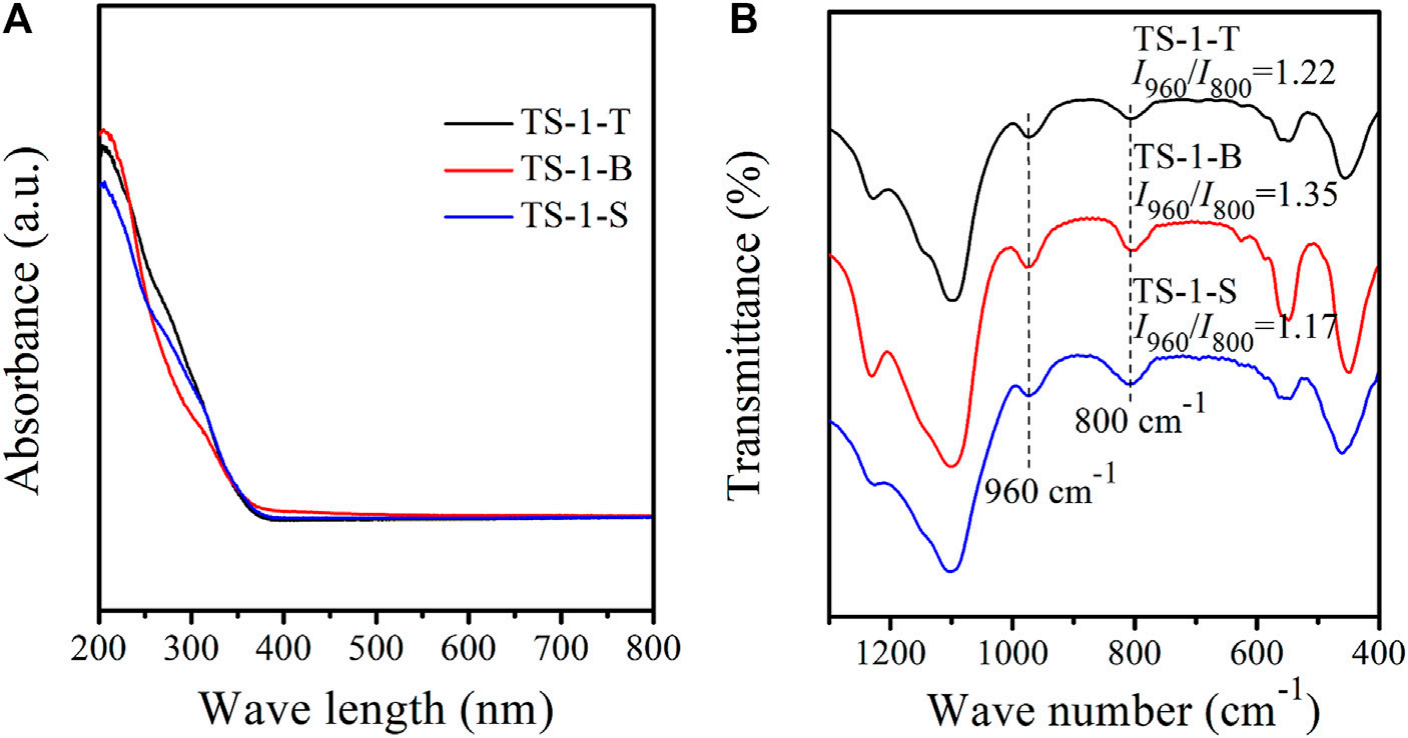
UV–Vis **(A)** and FT-IR **(B)** spectra of samples with titanium sources: TiCl_3_ (TS-1-T), TBOT (TS-1-B) and Ti(SO_4_)_2_ (TS-1-S).

UV–Vis spectroscopy is effective for detecting the coordination states of the Ti species in TS-1. Figure 2A shows the UV–Vis spectra of the three samples. The three adsorption bands at 210, 270, and 330 nm are ascribed to tetrahedral Ti (framework Ti) (Ricchiardi et al., 2001), extra-framework [HOTiO_3_] units (Balducci et al., 2003; Wells et al., 2004), and anatase TiO_2_ (Vayssilov, 1997), respectively. The intensities of the 270 and 330 nm bands in samples TS-1-T and TS-1-S were significantly higher than those in sample TS-1-B, suggesting higher amounts of extra-framework Ti and anatase TiO_2_ in the former two samples. Therefore, we concluded that the hydrolysis of the titanium and silicon sources were not well matched in this hydrothermal system when TiCl_3_ or Ti(SO_4_)_2_ were used, and the generation of extra-framework Ti and anatase TiO_2_ could be promoted by this incomplete hydrolysis.

Figure 2B shows the FT-IR spectra of these samples. All the three samples showed typical five-finger peaks, which correspond to the MFI topology (Astorino et al., 1995; Armaroli et al., 2001). The band at 800 cm^−1^ was ascribed to the absorption by the MFI structure (Naik et al., 2003). The band at 960 cm^−1^ is assigned to the stretching vibration of the Si-O bond perturbed by the neighboring framework Ti, which is regarded as evidence for the insertion of Ti atoms into the framework (Scarano et al., 1993). The intensity ratio of the peak at 960 cm^−1^ to that at 800 cm^−1^ (*I*_960_/*I*_800_) is often used to estimate the relative amount of framework Ti. As shown in Figure 2B, the *I*_960_/*I*_800_ of TS-1-B was higher than those of the other samples, indicating that TS-1-B contained the most framework Ti.

Supplementary Figure S3 shows the N_2_ sorption isotherms of the three samples. All samples showed similar type-I isotherms consistent with a microporous structure. Supplementary Table S4 shows the textural properties of these samples. Because of smaller crystal size and higher RC, the total surface area (*S*_BET_), micropore surface area (*S*_micro_), total pore volume (*V*_tot_), and micropore volume (*V*_micro_) of sample TS-1-B was slightly larger than those of others.

Table 1 shows the catalytic activities of these TS-1 samples towards propylene epoxidation. The framework Ti has been confirmed to be the active site for propylene epoxidation (Thangaraj et al., 1991). Consequently, sample TS-1-B with higher content of framework Ti and larger surface area showed higher catalytic activity than the other samples. Moreover, because the decomposition of H_2_O_2_ is promoted by a higher anatase TiO_2_ content (Khouw et al., 1994), the H_2_O_2_ utilization of sample TS-1-T and TS-1-S was 85.4 and 84.3%, respectively, which are lower than that of TS-1-B. These results demonstrate that TBOT was the optimal Ti source for the synthesis of TS-1 in the proposed system, as it facilitated the formation of TS-1 with a large amount of framework Ti and high catalytic activity. Hence, TBOT was used for further experiments.

**TABLE 1:** Catalytic activities towards propylene epoxidation of samples synthesized with different titanium sources.

Samples	Ti-source	*X*_H2O2_ (%)	*Y*_PO_ (%)	*S*_PO_ (%)	*U*_H2O2_ (%)
TS-1-T	TiCl_3_	76.1	63.2	97.2	85.4
TS-1-B	TBOT	81.2	70.8	96.5	90.4
TS-1-S	Ti(SO_4_)_2_	73.8	59.6	95.8	84.3

#### Si/Ti Molar Ratio

The catalytic activity of TS-1 is closely related to the tetrahedral Ti species in the framework (Thangaraj et al., 1991). Since Ti atoms are larger than Si atoms, and there are lattice limitations of the TS-1 zeolite structure, the maximum amount of Ti atoms inserted into the framework is 2.5 mol% (Millini et al., 1992), where additional Ti exists as anatase TiO_2_ or extra-framework Ti. The influence of the Si/Ti molar ratio on the properties of TS-1 was investigated by changing the amount of added TBOT. The obtained samples with *n* (Si/Ti) of 30, 35, 40, 45, 50, and 55 in the precursor sol are henceforth denoted as TS-1-30, TS-1-35, TS-1-40, TS-1-45, TS-1-B, and TS-1-55, respectively. The other synthesis conditions were the same: TBOT as the Ti source and a crystallization time of 24 h. The XRD diffractograms of these samples are shown in Supplementary Figure S4. All samples had pure MFI structures and high RC values (> 95%). The SEM and TEM images of these samples are presented in Supplementary Figures S5,S6, respectively. All samples showed a rounded-boat morphology (Roeffaers et al., 2008) with the crystal size of about 1 μm. Moreover, there is no significant difference in the N_2_ sorption isotherms (Supplementary Figure S7) and textural properties (Supplementary Table S5) of these samples.

**FIGURE 3:**
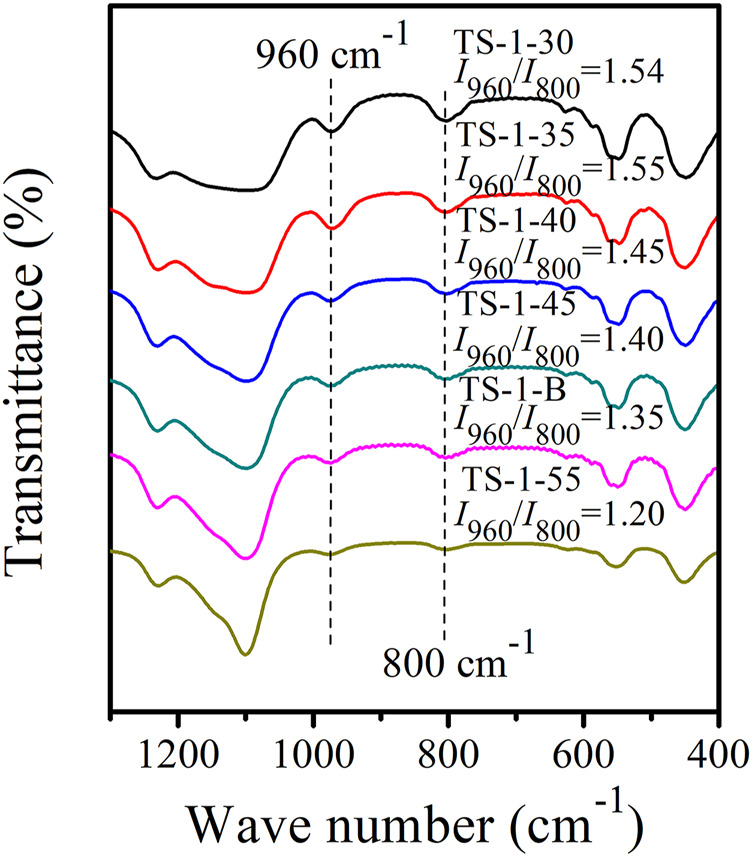
FT-IR spectra of samples with *n* (Si/Ti): 30 (TS-1-30), 35 (TS-1-35), 40 (TS-1-40), 45 (TS-1-45), 50 (TS-1-B), and 55 (TS-1-55).

Supplementary Table S6 summarizes the amounts of SiO_2_ and TiO_2_ in the bulk of the obtained solid zeolites. The molar ratios of Si/Ti in the bulk [*n*_B_(Si/Ti)] of the as-synthesized zeolites were slightly higher than those of the respective sols, may be owing to an increase in the Si content due to the introduction of the S-1 seeds. Supplementary Figure S8 shows the UV–Vis spectra of these samples. When *n*(Si/Ti) decreased from 55 to 35, the framework Ti content gradually increased, while the amounts of extra-framework Ti and anatase TiO_2_ hardly changed, suggesting that most of the additional Ti species entered the framework. However, when *n*(Si/Ti) further decreased to 30, the amount of framework Ti remained constant, and the additional Ti species contributed to slightly increasing the anatase TiO_2_ and extra-framework Ti. Figure 3 shows the FT-IR spectra and the *I*_960_/*I*_800_ for all the samples. The *I*_960_/*I*_800_ of sample TS-1-55 was only 1.20, and these values significantly increased with decreasing *n*(Si/Ti). When *n*(Si/Ti) decreased to 35, the maximum *I*_960_/*I*_800_ value was observed, indicating that this sample had the highest content of framework Ti. However, when the *n*(Si/Ti) further decreased to 30, the *I*_960_/*I*_800_ value remained unchanged, indicating that the content of framework Ti was saturated.

The catalytic activities of the samples towards propylene epoxidation were investigated (Table 2). All of the samples showed similar and high propylene oxide (PO) selectivity. When the *n* (Si/Ti) was varied from 55 to 35, the conversion of H_2_O_2_ increased remarkably, and sample TS-1-35 showed the highest catalytic activity owing to the maximum content of framework Ti. When the *n* (Si/Ti) was further reduced to 30, the H_2_O_2_ conversion was slightly high, while the PO yield and H_2_O_2_ utilization decreased remarkably due to the high content of anatase TiO_2_ accelerating the decomposition of H_2_O_2_ (Khouw et al., 1994).

**TABLE 2:** Catalytic activities towards propylene epoxidation of samples with different *n* (Si/Ti).

Sample	*n* (Si/Ti)	*X*_H2O2_ (%)	*Y*_PO_ (%)	*S*_PO_ (%)	*U*_H2O2_ (%)
TS-1-30	30	94.8	81.8	98.0	88.0
TS-1-35	35	93.2	84.1	97.1	92.9
TS-1-40	40	90.3	82.2	97.2	93.7
TS-1-45	45	85.4	77.0	97.0	93.0
TS-1-B	50	81.2	70.8	96.5	90.4
TS-1-55	55	75.9	67.5	97.3	91.4

#### Crystallization Time

The crystallization time plays a key role in the synthesis of TS-1. Furthermore, the addition of seeds to hydrothermal systems can decrease the energy barrier of crystallization and reduce the crystallization time (van der Pol and van Hooff, 1992; Chen et al., 2020). Therefore, the influence of the crystallization time was investigated with TBOT as the Ti source and *n* (Si/Ti) = 35 in the precursor sol.

**FIGURE 4:**
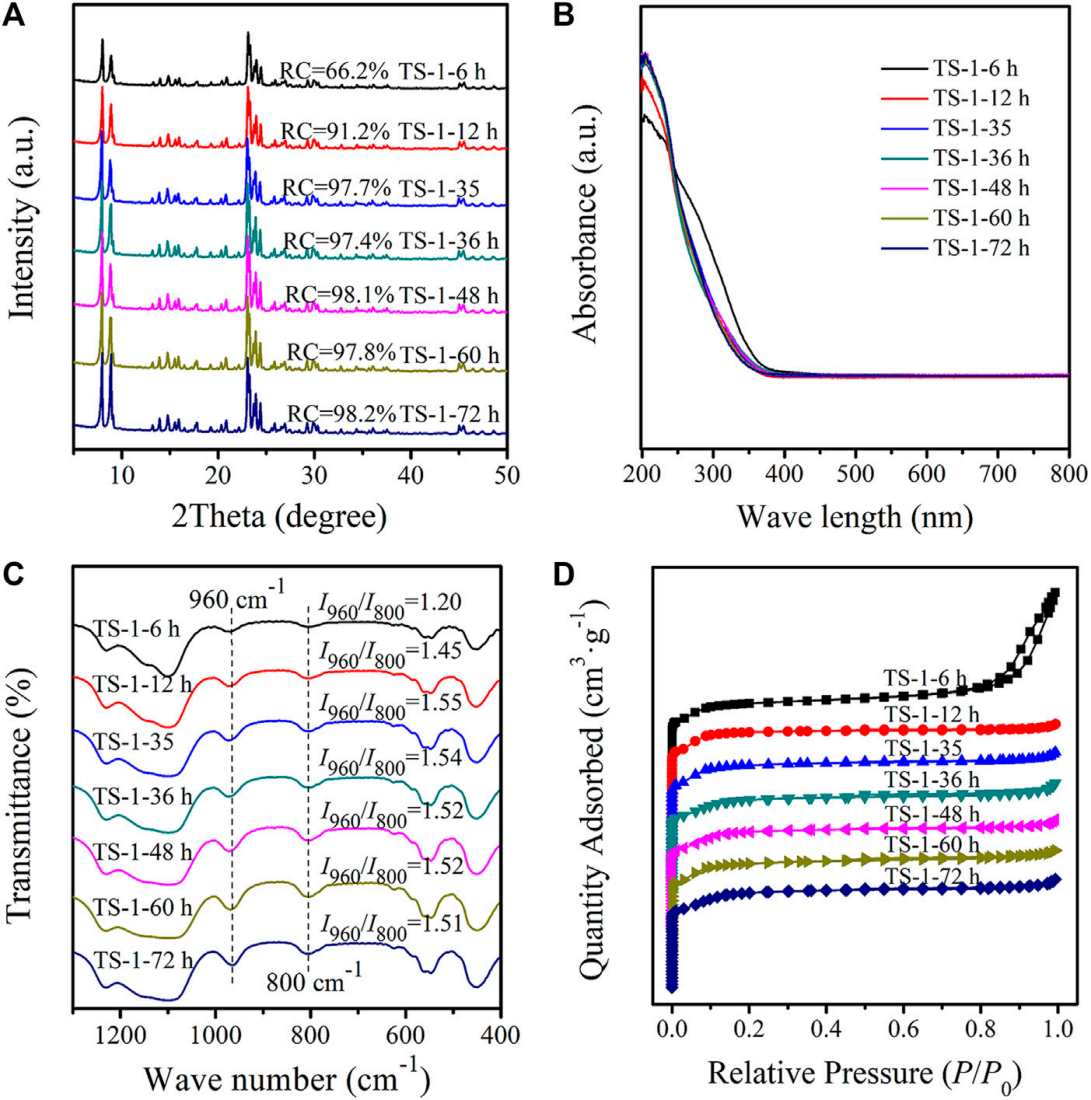
XRD diffractograms **(A)**, UV–Vis spectra **(B)**, FT-IR spectra **(C)**, and nitrogen sorption isotherms **(D)** of samples with crystallization time: 6 h (TS-1-6 h), 12 h (TS-1-12 h), 24 h (TS-1-35), 36 h (TS-1-36 h), 48 h (TS-1-48 h), 60 h (TS-1-60 h), and 72 h (TS-1-72 h).

XRD diffractograms of TS-1 samples with crystallization times of 6–72 h are presented in Figure 4A. All samples had characteristic peaks typical of the MFI structure, and a quite low RC value (66.2%) was obtained for a crystallization time of 6 h, suggesting that the MFI framework was not completely formed. As expected, the intensity of the diffraction peaks increased with a crystallization time of 12 h, and the RC of sample TS-1-12 h reached 91.2%. With further crystallization time, the RC increased continuously up to a processing time of 24 h, indicating that the crystallization was nearly complete at this time. The bulk chemical compositions of the obtained solid TS-1 zeolites are shown in Supplementary Table S7. With the increase in the degree of crystallization, the titanium content increased and *n*_B_(Si/Ti) decreased correspondingly. When crystallization was finally complete, the *n*_B_(Si/Ti) of the sample remained almost unchanged after 24 h.

A series of samples was synthesized without seeds for comparison, and the effect of the crystallization time on the RC was examined. The change in RC with increasing crystallization time of these samples is listed in Supplementary Figure S9. The RC values (˂70%) of the samples were quite low in the first 24 h of reaction time, which suggested that the crystallization was indeed faster in the presence of the nanosized S-1 seeds.

Figures 4B,C illustrate the UV–Vis and FT-IR (with the *I*_960_/*I*_800_ ratios) spectra of these samples with crystallization times of 6–72 h, respectively. TS-1-6 h had a rather low *I*_960_/*I*_800_ value of 1.20 and strong adsorption at the wavelengths of 270 and 330 nm in the UV–Vis spectra, which could be due to the incomplete MFI structure with few Ti atoms incorporated into the framework, with the remaining Ti forming anatase and extra-framework Ti. The gradual improvement in the framework structure enabled the amount of framework Ti to increase significantly. No significant differences in the analysis spectra were observed for crystallization times of more than 24 h, demonstrating that a stable MFI framework structure had been completely formed by this time. Without adding seeds, a sample was synthesized with a crystallization time of 72 h, and the UV–Vis and FT-IR spectra of this sample are presented in Supplementary Figures S10A,B respectively. Large amounts of extra-framework Ti and anatase were observed, and the *I*_960_/*I*_800_ value was only 1.20, which was much lower than that of sample TS-1-35 (processed for 24 h). Hence, the incorporation of Ti was effectively enhanced in the presence of seeds.

**FIGURE 5:**
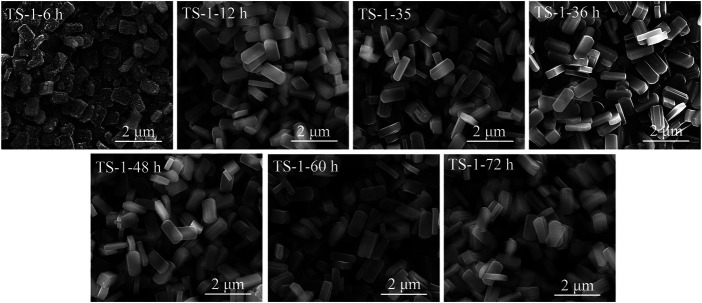
SEM images of samples with crystallization time: 6 h (TS-1-6 h), 12 h (TS-1-12 h), 24 h (TS-1-35), 36 h (TS-1-36 h), 48 h (TS-1-48 h), 60 h (TS-1-60 h), and 72 h (TS-1-72 h).

The SEM images of these samples are shown in Figure 5, and their crystal sizes are listed in Supplementary Table S8. All samples showed a rounded-boat morphology. Because of the incomplete crystallization, there were many amorphous phases on the surface of sample TS-1-6 h, which had a crystal size of 0.78 × 0.52 × 0.23 μm. When crystallization time was extended to 12 h, the crystal further grew to a size of 1.08 × 0.53 × 0.23 μm and had a smooth surface. The crystal grew in the direction of the *c*-axis (Roeffaers et al., 2008), suggesting a relatively slow crystallization rate of TS-1. The crystal sizes of the samples with crystallization times of more than 12 h did not significantly increase.

Figure 4D illustrates the N_2_ sorption isotherms of these samples. As the surface of sample TS-1-6 h was quite rough (Figure 5), inter-particle spaces were formed, which resulted in a pronounced hysteresis loop in the sorption isotherms, while the other samples showed similar type-I isotherms. Table 3 shows the textural properties of these samples. Owing to the inter-particle space, TS-1-6 h had the largest total pore volume. As expected, the total surface area, micropore surface area, and micropore volume slightly increased with increasing RC value. The samples produced with crystallization times of more than 24 h had similar textural properties.

**TABLE 3:** Textural properties of samples with different crystallization times.

Samples	Crystallization time	*S*_BET_	*S*_micro_	*V*_tot_	*V*_micro_
	H	m^2^/g		cm^3^/g		
TS-1-6 h	6	341	249	0.349	0.102
TS-1-12 h	12	401	317	0.198	0.126
TS-1-35	24	427	341	0.195	0.139
TS-1-36 h	36	428	347	0.192	0.143
TS-1-48 h	48	422	335	0.186	0.136
TS-1-60 h	60	425	337	0.200	0.137
TS-1-72 h	72	428	341	0.201	0.140

The catalytic activities towards propylene epoxidation of the samples prepared with different crystallization times were measured (Table 4). Owing to the lower RC and framework Ti content, TS-1-6 h showed poor catalytic activity towards propylene epoxidation. With increasing crystallization time, the RC and framework Ti content increased, resulting in a gradual increase in the catalytic activity of the zeolite. With a crystallization time of 24 h, TS-1-35 had a H_2_O_2_ conversion efficiency of 93.2% and PO yield of 84.1%. No further increase in the catalytic activity was observed for crystallization times longer than 24 h. Therefore, 24 h is sufficient to crystallize small-crystal TS-1.

**TABLE 4:** Catalytic activities towards propylene epoxidation of samples with different crystallization times.

Samples	Crystallization time (h)	*X*_H2O2_ (%)	*Y*_PO_ (%)	*S*_PO_ (%)	*U*_H2O2_ (%)
TS-1-6 h	6	60.5	51.1	93.5	90.3
TS-1-12 h	12	88.4	77.6	95.8	91.6
TS-1-35	24	93.2	84.1	97.1	92.9
TS-1-36 h	36	93.5	84.1	97.2	92.5
TS-1-48 h	48	94.1	83.3	96.9	91.4
TS-1-60 h	60	93.7	83.6	96.5	92.5
TS-1-72 h	72	94.2	83.5	97.4	91.0

Furthermore, TS-1-35 was modified with alkali to prepare the hierarchical TS-1 catalyst (denoted as HTS-1) for HPPO (Liu et al., 2018). Supplementary Figure S11 shows a TEM image of HTS-1. As a result of the desilication and recrystallization, coffin-shaped crystals containing many hollows were produced. In addition, the molar ratio of Si/Ti on the surface [*n*_S_(Si/Ti)] was much higher than that in the bulk [*n*_B_(Si/Ti)] (see Supplementary Table S9), indicating a high surface hydrophobicity (Dai et al., 2017). For comparison, nanosized TS-1 (denoted NTS-1) was synthesized using a standard expensive template (TPAOH). The chemical composition of this sample is also shown in Supplementary Table S9. The *n*_B_(Si/Ti) values of NTS-1 and HTS-1 were quite similar; however, the *n*_S_(Si/Ti) of NTS-1 was much lower than that of sample HTS-1, which indicates the relatively poor surface hydrophobicity of NTS-1 (Dai et al., 2017). The HTS-1 and NTS-1 catalysts were then used for propylene epoxidation, and the results are shown in Supplementary Table S10. Notably, owing to the elimination of the diffusion resistance by the hierarchical structure and the enhanced adsorption of propylene provided by the high surface hydrophobicity (Wang et al., 2019), HTS-1 had an extremely high TOF of 1,650 h−^1^ based on H_2_O_2_, which was much higher than that of NTS-1 sample and those of TS-1 samples in our previous work (Liu et al., 2016) and other literatures (Zuo et al., 2012; Xiong et al., 2018; Li et al., 2019; Wang et al., 2019) (seen in Supplementary Table S11), indicating the high catalytic efficiency of hierarchical small-crystal TS-1.

### Green Synthesis of Titanium Silicalite-1

The findings of this study indicated that the optimized synthesis conditions are: TBOT as the Ti source, *n* (Si/Ti) = 35 in the precursor sol, and a crystallization time of 24 h. The residual concentrations of EOA and TPABr in the obtained mother liquid were approximately 0.22 and 0.03 mol/L, respectively (detailed analysis in Supplementary Material). To evaluate the potential for green synthesis of small-crystal TS-1, where the mother liquid is completely recycled, the influence of the EOA and TPABr concentrations during the mother liquid recycling process (MLRP) on the final products were systematically studied.

#### Ethanolamine Content

**FIGURE 6:**
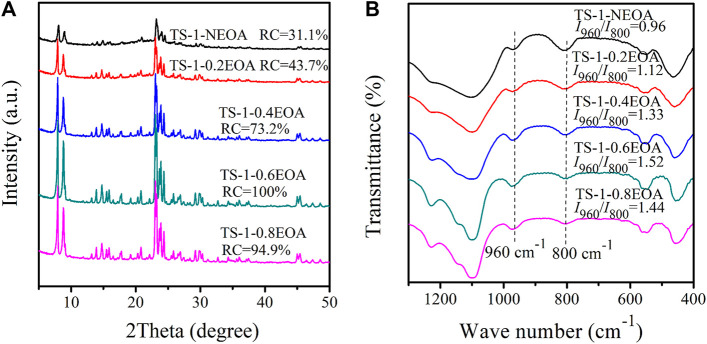
XRD diffractograms **(A)** and FT-IR spectra **(B)** of samples synthesized with various EOA contents in the MLRP: without EOA (TS-1-NEOA), 0.20 mol/L (TS-1-0.2EOA), 0.40 mol/L (TS-1-0.4EOA), 0.60 mol/L (TS-1-0.6EOA), and 0.80 mol/L (TS-1-0.8EOA).

It has been shown that alkalis act as mineralizing agents in hydrothermal synthesis processes (Cundy and Cox, 2005). The silicon and titanium in raw materials are dissolved by the alkali and then crystallize in the presence of the template material. The alkalinity of the hydrothermal system plays a vital role in the hydrolysis and polymerization of silicon and titanium. Figure 6A shows the XRD diffractograms of synthesized samples with different amounts of EOA in MLRP. Even without EOA, the obtained TS-1-NEOA also had a typical MFI topology structure, indicating that a certain amount of alkali remained in the mother liquid. However, the RC of sample TS-1-NEOA was only 31.1% (Figure 6A) due to the insufficient alkalinity to dissolve the silicon, leading to the formation of amorphous compounds (van der Pol and van Hooff, 1992; Wang et al., 2012). With higher EOA content, the alkalinity increased, and the RC value of the corresponding sample was gradually improved to 100%. Nevertheless, when the EOA content was increased to 0.8 mol/L, the silicon and titanium would also be strongly dissolved, limiting their crystallization. Consequently, the RC of TS-1-0.8EOA decreased to 94.9%. Moreover, with increasing EOA content, the bulk Ti content exhibited a similar trend to that of the RC (Supplementary Table S12).

The SEM and TEM images of these samples are shown in Supplementary Figures S12,S13, respectively. Because of lower RCs, a large amount of amorphous compounds can be clearly observed on the surfaces of samples TS-1-NEOA and TS-1-0.2EOA. With the increase of EOA content, the resultant samples showed similar rounded-boat morphology and smooth surface to that of the freshly synthesized TS-1-35 sample (without MLRP). The N_2_ sorption isotherms of these samples are illustrated in Supplementary Figure S14. Due to the rough surfaces, the sorption isotherms of samples TS-1-NEOA and TS-1-0.2EOA showed small hysteresis loops at a high relative pressure (*P*/*P*_0_ > 0.9) caused by inter-particle spaces. The textural properties of these samples are summarized in Supplementary Table S13. Because of higher RC, the total surface area, micropore surface area, and micropore volume of sample TS-1–0.6EOA was slightly larger than those of others. While, TS-1-NEOA and TS-1-0.2EOA had larger total pore volume, which was ascribed to the inter-particle spaces.

The FT-IR spectra and *I*_960_/*I*_800_ values of all samples are shown in Figure 6B. Sample TS-1-0.6EOA clearly had the highest content of framework Ti. Large EOA contents promote the formation of silicate oligomers, which could promote the incorporation of Ti atoms into the framework (Wang et al., 2012). For EOA contents above 0.60 mol/L, the content of framework Ti slightly decreased.

Table 5 shows the catalytic activities of the samples towards propylene epoxidation. As the EOA content gradually increased, the catalytic activity of the samples increased, where TS-1-0.6EOA had the best overall catalytic performance with the highest H_2_O_2_ conversion (94.2%) and PO yield (85.2%). When the EOA content further increased to 0.80 mol/L, a decrease in the catalytic activity of TS-1-0.8EOA was observed, probably due to the decrease in the amount of framework Ti and RC. Therefore, 0.6 mol/L was considered the optimal EOA content in the MLRP for the studied system, which ensured the optimal catalytic performance of the product and a 25% savings in EOA usage.

**TABLE 5:** Catalytic activities towards propylene epoxidation of samples synthesized with various EOA contents in the MLRP.

Samples	Amount of EOA (mol/L)	*X*_H2O2_ (%)	*Y*_PO_ (%)	*S*_PO_ (%)	*U*_H2O2_ (%)
TS-1-NEOA	0	45.7	39.8	96.9	89.9
TS-1-0.2EOA	0.20	77.5	69.1	97.4	91.5
TS-1-0.4EOA	0.40	87.5	79.0	97.7	92.4
TS-1-0.6EOA	0.60	94.2	85.2	97.2	93.1
TS-1-0.8EOA	0.80	91.2	81.3	97.3	91.6

#### Tetrapropylammonium Bromide Content

**FIGURE 7:**
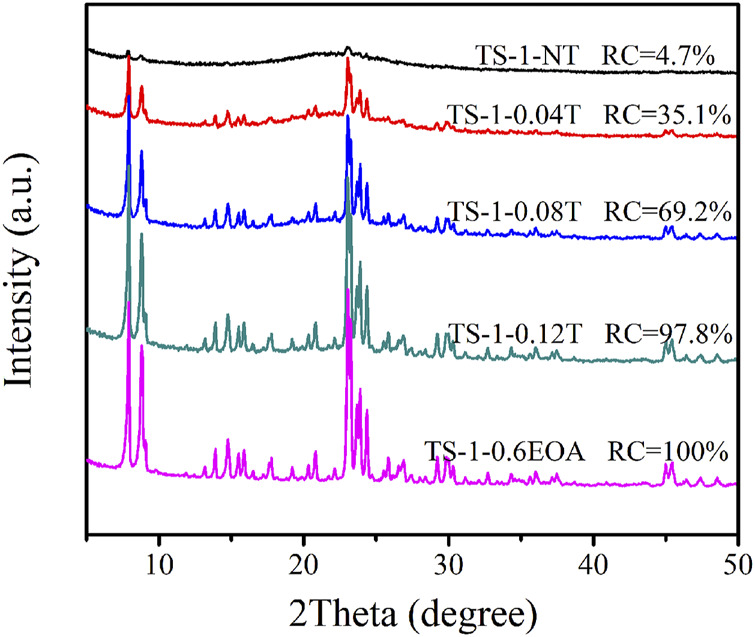
XRD diffractograms of samples synthesized with various TPABr contents in the MLRP: without TPABr (TS-1-NT), 0.04 mol/L (TS-1-0.04T), 0.08 mol/L (TS-1-0.08T), 0.12 mol/L (TS-1-0.12T), and 0.16 mol/L (TS-1-0.6EOA).

The template plays a crucial role in the crystallization of zeolites. Silicon and titanium in the precursor can crystallize in the presence of the template material and further grow into zeolite crystals. A crystal cell of TS-1 contains four TPA^+^, and the minimal molar ratio of TPA^+^/SiO_2_ is 0.042 (Tuel, 1996). Figure 7 illustrates the XRD diffractograms of samples synthesized with different TPABr contents in the MLRP. When no TPABr was added, the obtained sample (TS-1-NT) did not show characteristic diffraction peaks of the MFI topology and had a low RC of 4.7%, indicating that little TPABr remained in the mother liquid. With increasing TPABr content, more silicon and titanium crystallized, indicated by the strengthened characteristic peaks of the MFI structure and the higher RC values. However, when the TPABr exceeded 0.12 mol/L, the RC did not change further. In addition, almost no titanium species were detected in TS-1NT. The titanium content of the obtained samples exhibited a similar trend to that of the RC with increasing TPABr content (see Supplementary Table S14).

Supplementary Figure S15 presents the SEM images of the samples prepared with different TPABr contents. TS-1-NT was entirely amorphous, and no TS-1 particles were observed, which is consistent with the XRD results. With the increase in the amount of the template, the RC of the obtained sample gradually increased, and obvious TS-1 crystals with rounded-boat morphology were observed. When the TPABr content reached 0.12 mol/L, TS-1-0.12T showed a smooth surface with a crystal size of approximately 1.06 × 0.52 × 0.23 μm. Further increasing the TPABr content did not change the morphology of the obtained sample.

Supplementary Figure S16 shows the nitrogen sorption isotherms of these samples. TS-1-NT showed a large hysteresis loop at a high relative pressure (*P*/*P*_0_ > 0.9), probably due to the presence of inter-crystalline voids caused by the amorphous compounds. With increasing TPABr content, the amounts of amorphous compounds gradually decreased, resulting in reduced size of the hysteresis loop. When the TPABr content reached 0.12 and 0.16 mol/L, the obtained samples showed type-I isotherms, similar to that of the fresh TS-1-35. The textural properties of these samples are summarized in Supplementary Table S15. As expected, TS-1-0.12T and TS-1-0.6EOA had the largest total surface areas, micropore surface areas, and micropore volumes.

In addition, the catalytic activities of these samples towards propylene epoxidation were investigated (Table 6). The H_2_O_2_ conversion increased significantly with increasing TPABr content. Owing to the larger surface areas and higher RC values, TS-1-0.12T and TS-1-0.6EOA had the highest catalytic activities, similar to that of fresh TS-1-35, confirming the feasibility of synthesizing these materials using the proposed MLRP. Therefore, we concluded that the optimum amount of TPABr to guarantee complete crystallization of the zeolite was 0.12 mol/L in the MLRP, which was 75% of that used in the first synthesis.

**TABLE 6:** Catalytic activities towards propylene epoxidation of samples synthesized with various TPABr contents in the MLRP.

Samples	Amount of TPABr (mol/L)	*X*_H2O2_ (%)	*Y*_PO_ (%)	*S*_PO_ (%)	*U*_H2O2_ (%)
TS-1-NT	0	4.7	3.8	95.8	84.4
TS-1-0.04T	0.04	36.9	31.5	96.7	88.3
TS-1-0.08T	0.08	69.0	60.4	97.2	90.1
TS-1-0.12T	0.12	93.8	85.0	97.5	92.9
TS-1-0.6EOA	0.16	94.2	85.2	97.2	93.1

#### Sustainable Recycling of the Mother Liquid

To achieve the sustainable green synthesis of TS-1 with little wastewater, the mother liquid produced in each batch must be used for the next synthesis procedure. Thus, continuous mother liquid recycling was performed under the conditions described above. We reused the mother liquid a total of ten times, and the resulting samples were denoted as TS-1-Rn (n indicates the number of cycles, Table 7). There was no obvious difference in the appearance, pore structure, RC, coordination state of the Ti species, or chemical composition of all samples (Supplementary Figures S17–S21, Supplementary Tables S16,S17), indicating that the synthesis of TS-1 via the MLRP strategy is feasible. In addition, Supplementary Table S18 shows the yield of zeolite for each batch based on the amounts of SiO_2_ and TiO_2_ in the precursor sol. The zeolite yield was increased during the recycling process, probably because the unutilized silica and titanium in the mother liquid acted as additional precursors for synthesizing the TS-1 zeolite. Notably, almost no wastewater was discharged during the entire process, and the residual concentrations of EOA and TPABr in the final batch of mother liquid were approximately 0.13 and 0.02 mol/L, respectively. The utilization ratios of both EOA and TPABr were greatly increased to more than 95% (Supplementary Equations S5,S6). As expected, all samples demonstrated catalytic activities towards propylene epoxidation similar to that of the fresh sample (Table 7).

**TABLE 7:** Catalytic activities towards propylene epoxidation of samples synthesized by sustainably recycling mother liquid.

Samples	Mother liquid recycling	*X*_H2O2_ (%)	*Y*_PO_ (%)	*S*_PO_ (%)	*U*_H2O2_ (%)
TS-1-35	–	93.2	84.1	97.1	92.9
TS-1-R1	From 1st batch	93.8	85.0	97.5	92.9
TS-1-R2	From 2nd batch	96.7	86.8	97.5	92.0
TS-1-R3	From 3rd batch	93.6	83.6	97.2	91.9
TS-1-R4	From 4th batch	96.5	85.9	97.5	91.3
TS-1-R5	From 5th batch	95.0	83.1	96.6	90.6
TS-1-R6	From 6th batch	96.0	85.3	97.5	91.1
TS-1-R7	From 7th batch	95.5	85.6	96.8	92.6
TS-1-R8	From 8th batch	94.8	87.0	98.1	93.5
TS-1-R9	From 9th batch	95.3	85.2	97.5	91.7
TS-1-R10	From 10th batch	94.5	85.8	97.8	92.8

## Conclusion

In this study, we proposed a novel method for the hydrothermal synthesis of TS-1 zeolite in a TPABr-ethanolamine system and analyzed the effect of synthesis conditions on its catalytic activity in detail. The optimized TS-1 had the highest amount of framework Ti and RC. Moreover, the crystallization time was decreased to 24 h with the addition of nanosized S-1 seeds to the hydrothermal solution. Using this TS-1 zeolite as the precursor, the obtained hierarchical HTS-1 catalyst showed a high TOF value of 1,650 h^−1^ towards propylene epoxidation, which was attributed to the reduced diffusion barrier in the hierarchical structure and the favorable adsorption of propylene due to the high surface hydrophobicity. Unlike conventional methods for synthesizing TS-1 zeolite that produce large quantities of wastewater, we demonstrated that the mother liquid could be recycled ten times, resulting in very high utilization ratios of the precursors and no catalytic performance degradation of the produced zeolite. Hence, this method has the potential to be applied as a green and sustainable process for synthesizing high-performance TS-1 and other conventional zeolites and is of theoretical significance and practical value.

## Data Availability

The original contributions presented in the study are included in the article/Supplementary Material, further inquiries can be directed to the corresponding authors.
