# Supplementary material for: Synthesis of Low Cost Titanium Silicalite-1 Zeolite for Highly Efficient Propylene Epoxidation
Source: Front Chem. 2021 Jun 7;9:682404. doi: 10.3389/fchem.2021.682404 (PMC8215215; doi:10.3389/fchem.2021.682404)
Supplement: Supplementary file 1 [file DataSheet1.docx]

Supplementary Material

Supplementary Data

***Determination of the concentrations of EOA and TPABr in the filtrate***

The concentration of ethanolamine in the filtrate was analyzed by using a GC 9790 plus gas chromatograph equipped with an FID detector and a KB-624 capillary column (60 m × 0.25 mm × 0.33 μm). The concentrations of TPABr in the filtrate were analyzed by using an Agilent 1200 HPLC equipped with an ultraviolet detector (λ = 210 nm) and a ZORBAX SB-CN column (4.5 μm, 150 mm × 4.6 mm).

***Analysis of reaction results***

Iodometric titration was used to measure the residual H_2_O_2_. The gas chromatograph (GC 9790 plus) with an FID detector and a KB-624 capillary column (60 m × 0.25 mm × 0.33 μm) was employed to detect the products. The main product was propylene oxide (PO), and the main byproducts were propylene glycol (PG) and propylene glycol monomethyl ethers (MME). The conversion of H_2_O_2_ (*X*_H2O2_), yield of PO (*Y*_PO_), selectivity to PO (*S*_PO_), and utilization of H_2_O_2_ (*U*_H2O2_) were calculated according to following formula:

The conversion of H_2_O_2_:

*X*_H2O2_ = (*n*_0_(H_2_O_2_) － *n*(H_2_O_2_))/*n*_0_(H_2_O_2_) × 100% Eqution S1

The yield of PO:

*Y*_PO_ = *n*(PO)/*n*_0_(H_2_O_2_) × 100% Eqution S2

The selectivity to PO:

*S*_PO_ = *n*(PO)/(*n*(PO) ＋ *n*(MME) ＋ *n*(PG)) × 100% Eqution S3

The utilization of H_2_O_2_:

*U*_H2O2_ = (*n*(PO) ＋ *n*(MME) ＋ *n*(PG))/(*n*_0_(H_2_O_2_) × *X*_H2O2_) × 100% Eqution S4

The *n*_0_(H_2_O_2_) and *n*(H_2_O_2_) are the initial and final molar number of H_2_O_2_, respectively. The *n*(PO), *n*(PG) and *n*(MME) are the number of molar of PO, PG, and MME, respectively.

***Characterizations***

The X-ray powder diffraction (XRD) pattern was recorded by a PW 3040/60 diffractometer using Cu Kα radiation (λ = 1.54 Å). Fourier transform infrared (FT-IR) spectra were obtained on a Nicolet Nexus 470 FT-IR instrument, and the KBr pellet technique was used. Ultraviolet-visible (UV-vis) spectra were recorded with a spectrometer (Agilent Cary 5000) by employing BaSO_4_ as reference. The amounts of SiO_2_ and TiO_2_ of obtained zeolites in the bulk were measured by a Thermo Scientific iCAP6000 ICP Spectrometer. The samples were digested by 40 wt.% NaOH solution at 175 °C in a Teflon-lined autoclave for 24 h to determine the content of Si, and the samples were digested by 40 wt.% HF solution to determine the content Ti. The amounts of SiO_2_ and TiO_2_ of obtained zeolites on the surface were measured by a Thermon ESCAlab 250 X-ray photoelectron spectrometer (USA) using Al Kα radiation. Nitrogen sorption measurements were performed using a Micromeritics ASAP 2020 instrument at 77 K. Total surface area (*S*_BET_) was determined through the BET method. The amount adsorbed at the relative pressure *P*/*P*_0_ of 0.99 was applied to determine the total pore volume (*V*_tot_). The *t*-plot method was used to calculate micropore surface area (*S*_micro_) and micropore volume (*V*_micro_). The appearance of TS-1 was imaged on a scanning electron microscope (SEM, Quanta FEG 250) and a transmission electron microscope (TEM, JEM-2100).

***Formulas for utilizations of ethanolamine (U_EOA_) and TPABr (U_TPABr_)***

The utilizations of ethanolamine (U_EOA_) and TPABr (U_TPABr_) were calculated as follows:

*U*_EOA_ = (*n*_0_(EOA) － *n*(EOA))/*n*_0_(EOA) × 100% Eqution S5

*U*_TPABr_ = (*n*_0_(TPABr) － *n*(TPABr))/*n*_0_(TPABr) × 100% Eqution S6

The *n*_0_(EOA) and *n*_0_(TPABr) represent the added amount of ethanolamine and TPABr in the whole process, respectively. The *n*(EOA) and *n*(TPABr) represent the residual amount of ethanolamine and TPABr in the mother liquid produced in the final batch, respectively.

# Supplementary Figures and Tables

# *Supplementary Figures*


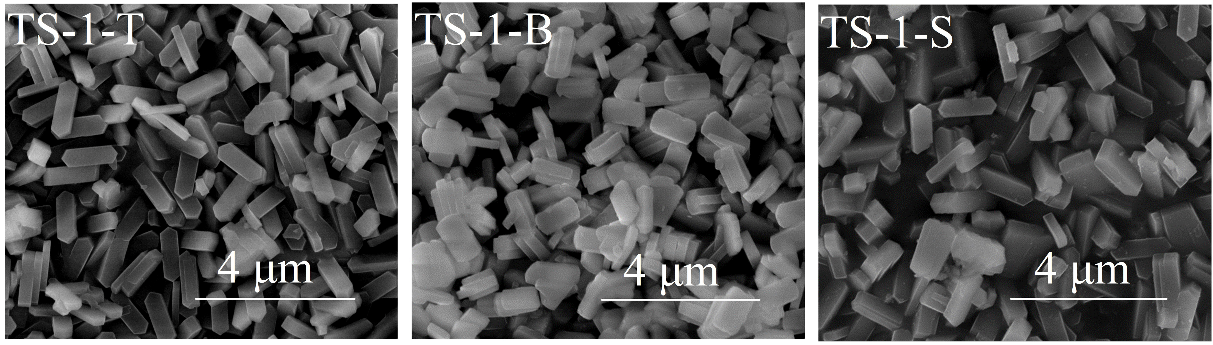


**Supplementary Figure S1.** SEM images of samples with titanium source: TiCl_3_ (TS-1-T), TBOT (TS-1-B), and Ti(SO_4_)_2_ (TS-1-S).


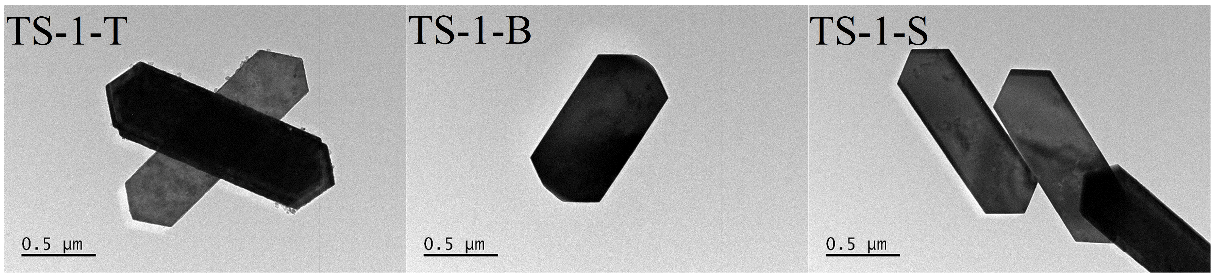


**Supplementary Figure S2.** TEM images of samples with titanium source: TiCl_3_ (TS-1-T), TBOT (TS-1-B), and Ti(SO_4_)_2_ (TS-1-S).


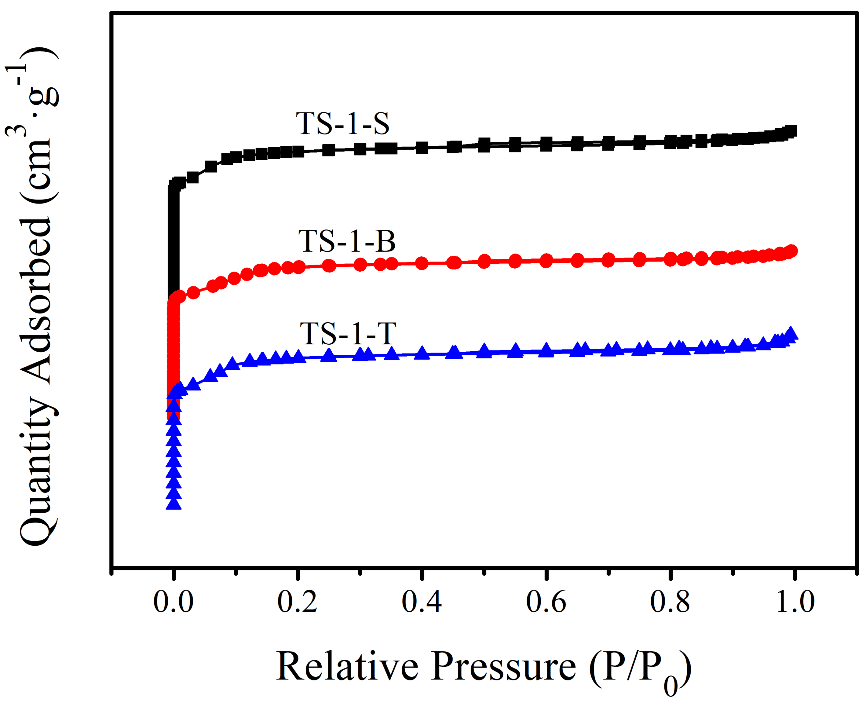


**Supplementary Figure S3.** Nitrogen sorption isotherms of samples with titanium source: TiCl_3_ (TS-1-T), TBOT (TS-1-B), and Ti(SO_4_)_2_ (TS-1-S).


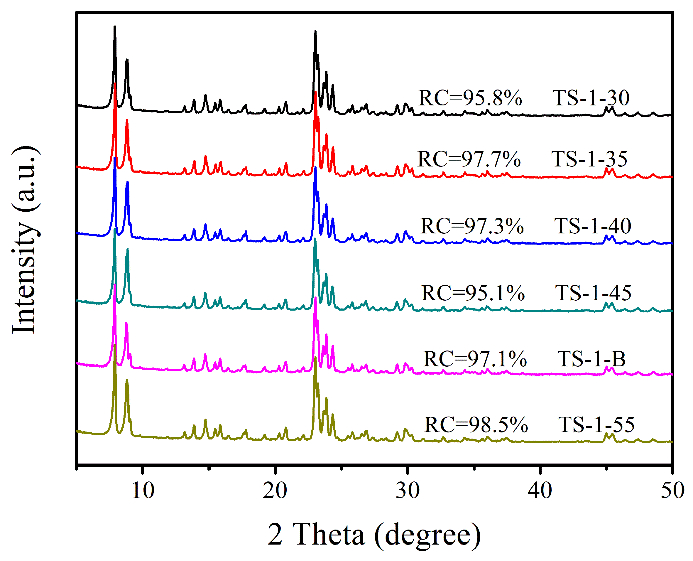


**Supplementary Figure S4.** XRD diffractograms of samples with *n*(Si/Ti): 30 (TS-1-30), 35 (TS-1-35), 40 (TS-1-40), 45 (TS-1-45), 50 (TS-1-B) and 55 (TS-1-55).


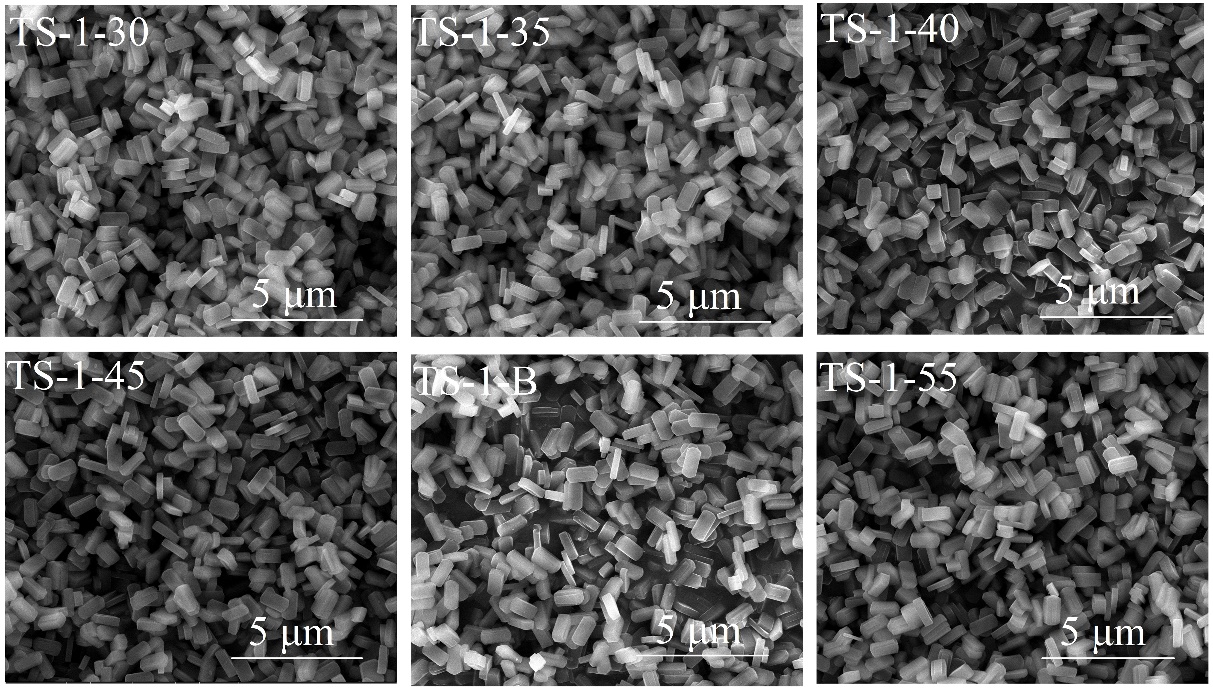


**Supplementary Figure S5.** SEM images of samples with *n*(Si/Ti): 30 (TS-1-30), 35 (TS-1-35), 40 (TS-1-40), 45 (TS-1-45), 50 (TS-1-B) and 55 (TS-1-55).


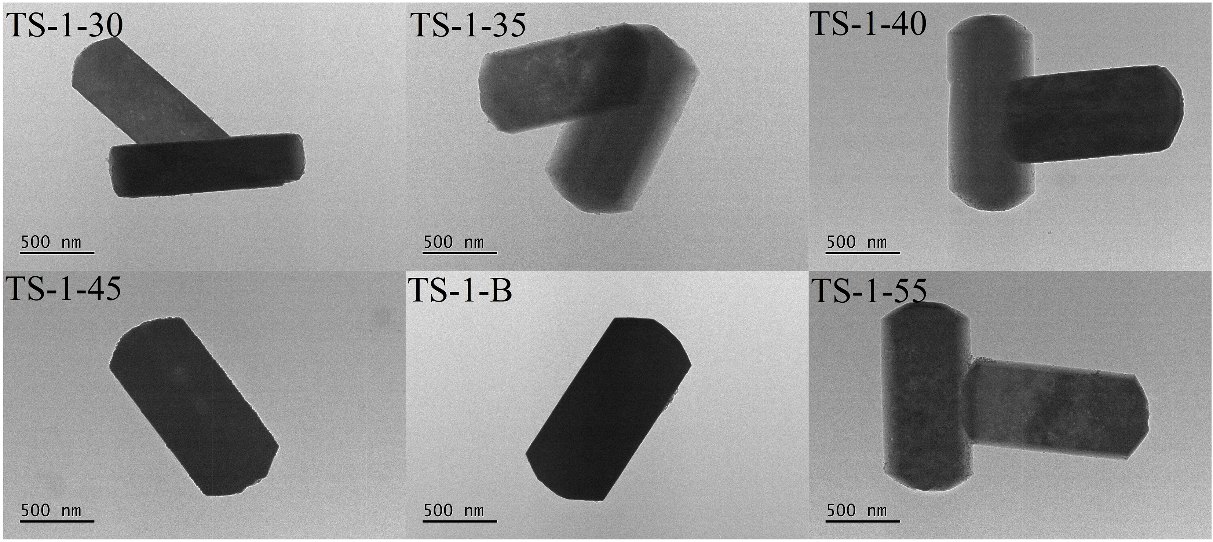


**Supplementary Figure S6.** TEM images of samples with *n*(Si/Ti): 30 (TS-1-30), 35 (TS-1-35), 40 (TS-1-40), 45 (TS-1-45), 50 (TS-1-B) and 55 (TS-1-55).

**
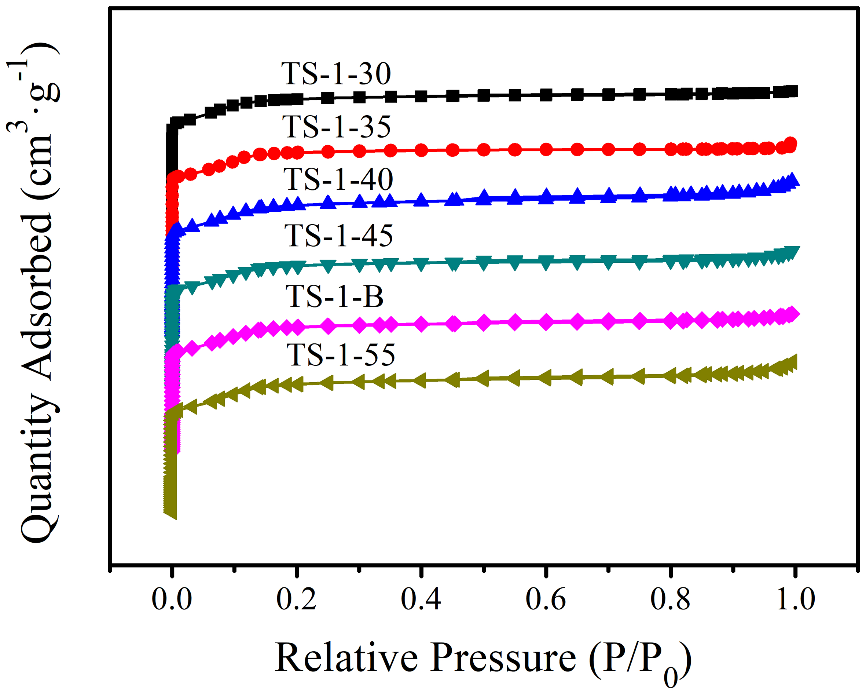
**

**Supplementary Figure S7.** Nitrogen sorption isotherms of samples with *n*(Si/Ti): 30 (TS-1-30), 35 (TS-1-35), 40 (TS-1-40), 45 (TS-1-45), 50 (TS-1-B) and 55 (TS-1-55).

**
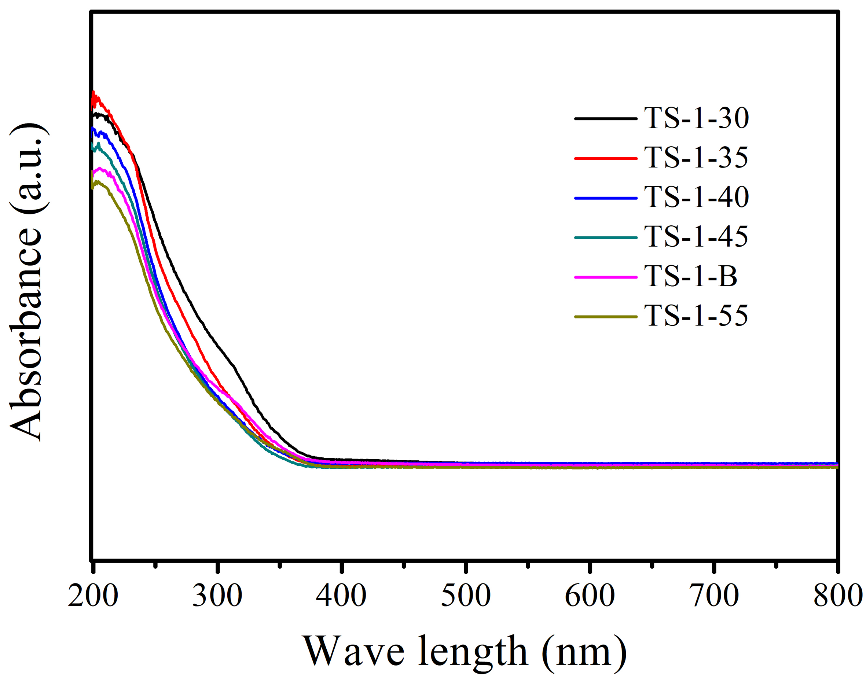
**

**Supplementary Figure S8.** UV–vis spectra of samples with *n*(Si/Ti): 30 (TS-1-30), 35 (TS-1-35), 40 (TS-1-40), 45 (TS-1-45), 50 (TS-1-B) and 55 (TS-1-55).


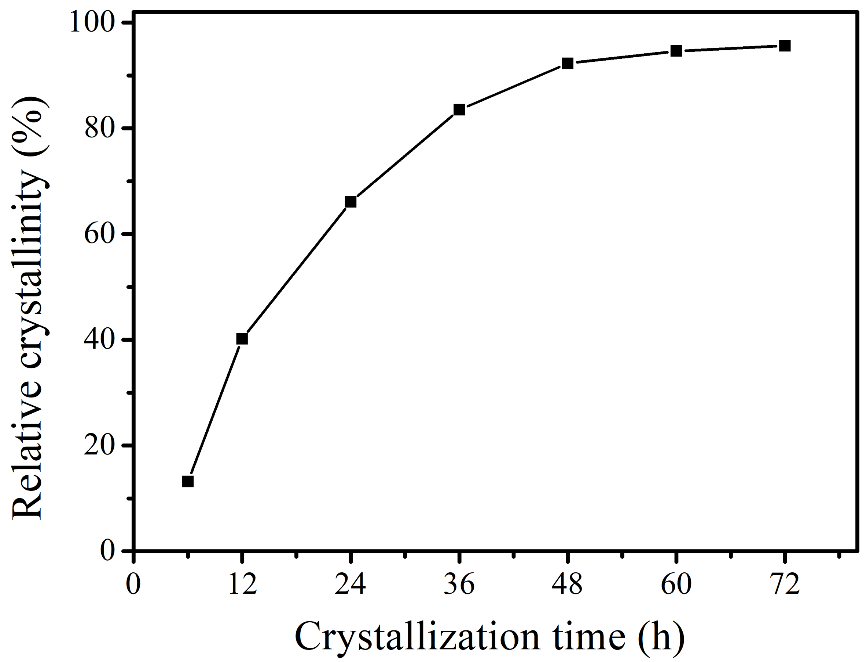


**Supplementary Figure S9.** The crystallization curve of samples synthesized without adding seed.


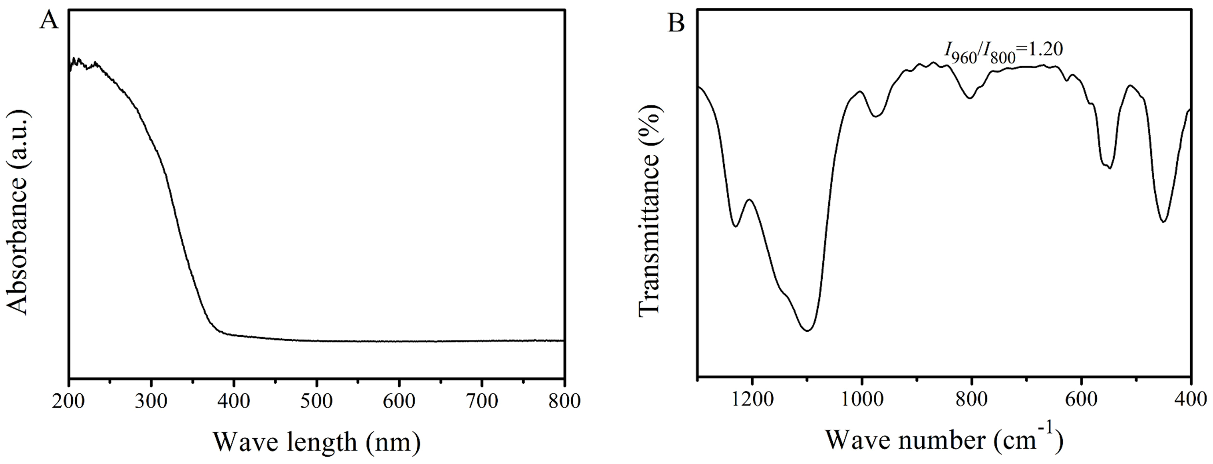


**Supplementary Figure S10.** UV–vis **(A)** and FT-IR **(B)** spectra of sample synthesized without adding seed.


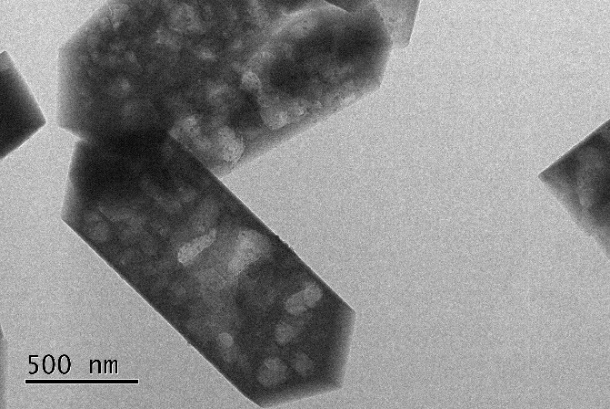


**Supplementary Figure S11.** TEM images of sample HTS-1.


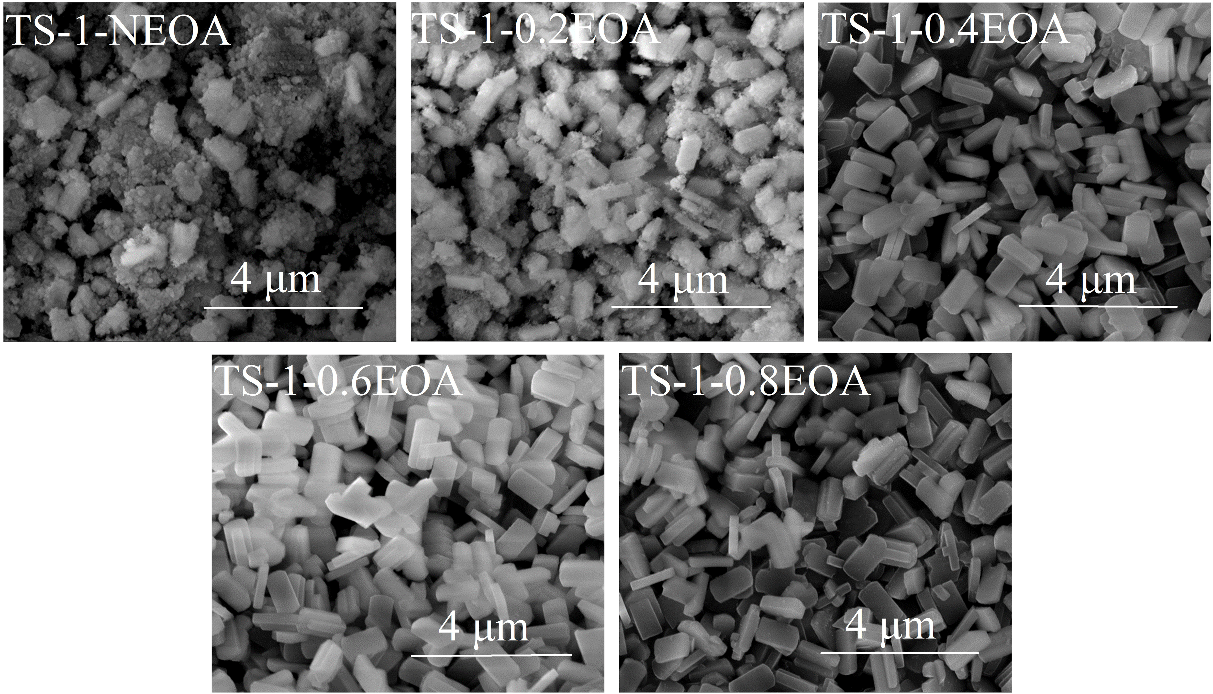


**Supplementary Figure S12.** SEM images of TS-1 samples synthesized with various EOA contents in the MLRP: without EOA (TS-1-NEOA), 0.20 mol/L (TS-1-0.2EOA), 0.40 mol/L (TS-1-0.4EOA), 0.60 mol/L (TS-1-0.6EOA), and 0.80 mol/L (TS-1-0.8EOA).


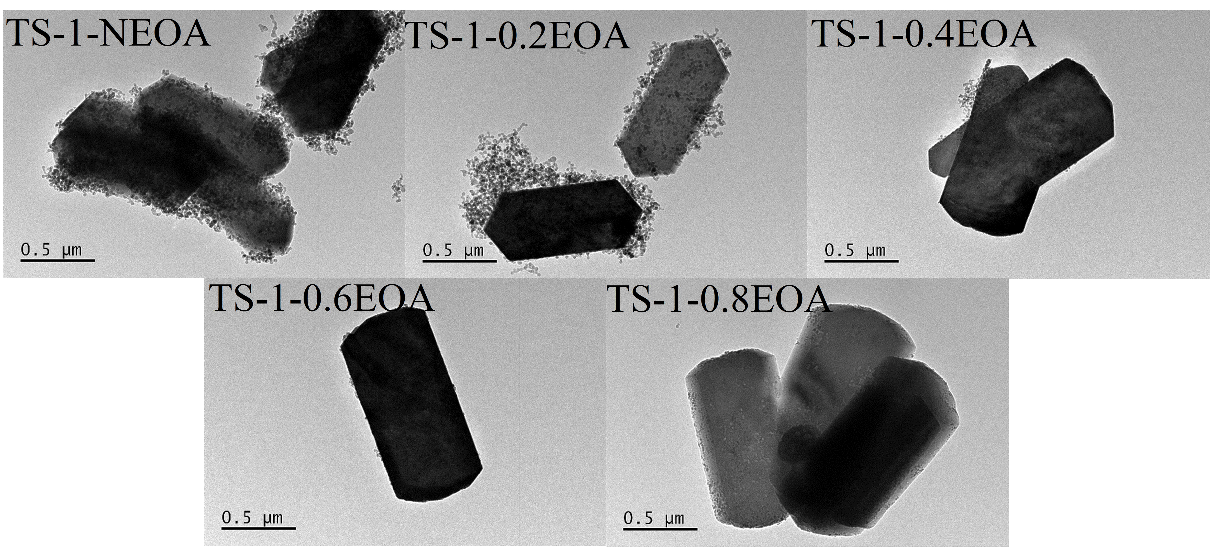


**Supplementary Figure S13.** TEM images of TS-1 samples synthesized with various EOA contents in the MLRP: without EOA (TS-1-NEOA), 0.20 mol/L (TS-1-0.2EOA), 0.40 mol/L (TS-1-0.4EOA), 0.60 mol/L (TS-1-0.6EOA), and 0.80 mol/L (TS-1-0.8EOA).


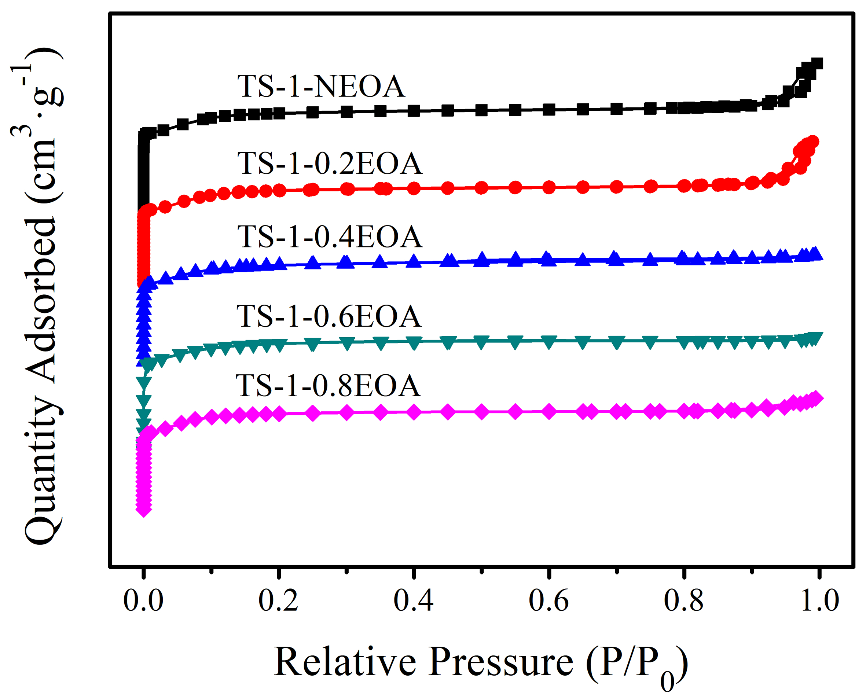


**Supplementary Figure S14.** Nitrogen sorption isotherms of TS-1 samples synthesized with various EOA contents in the MLRP: without EOA (TS-1-NEOA), 0.20 mol/L (TS-1-0.2EOA), 0.40 mol/L (TS-1-0.4EOA), 0.60 mol/L (TS-1-0.6EOA), and 0.80 mol/L (TS-1-0.8EOA).


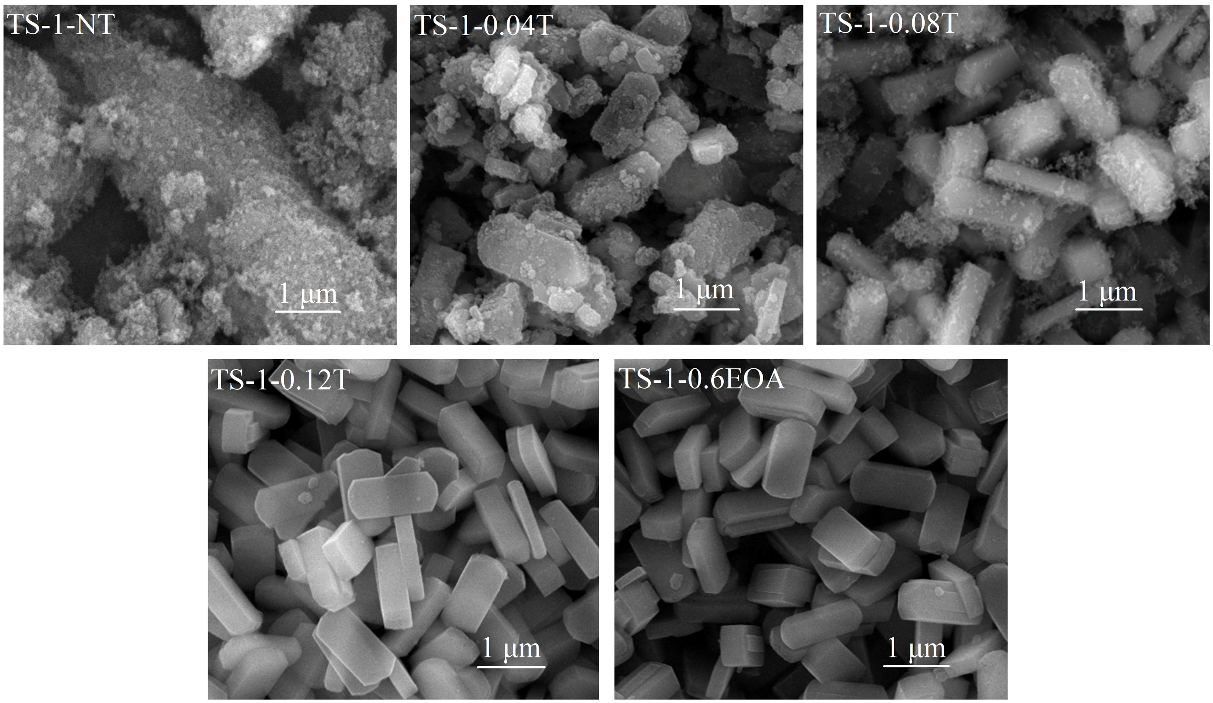


**Supplementary Figure S15.** SEM images of TS-1 samples synthesized with various TPABr contents in the MLRP: without TPABr (TS-1-NT), 0.04 mol/L (TS-1-0.04T), 0.08 mol/L (TS-1-0.08T), 0.12 mol/L (TS-1-0.12T), 0.16 mol/L (TS-1-0.6EOA).


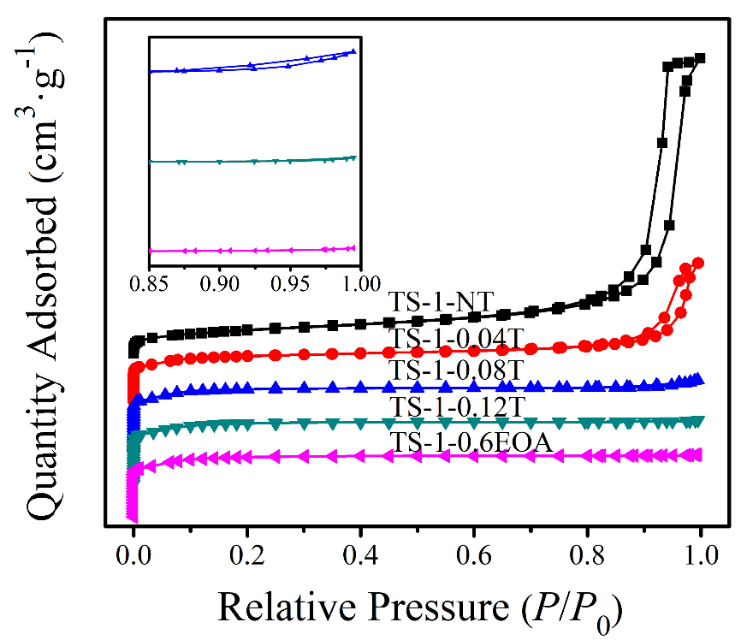


**Supplementary Figure S16.** Nitrogen sorption isotherms of TS-1 samples synthesized with various TPABr contents in the MLRP: without TPABr (TS-1-NT), 0.04 mol/L (TS-1-0.04T), 0.08 mol/L (TS-1-0.08T), 0.12 mol/L (TS-1-0.12T), 0.16 mol/L (TS-1-0.6EOA).


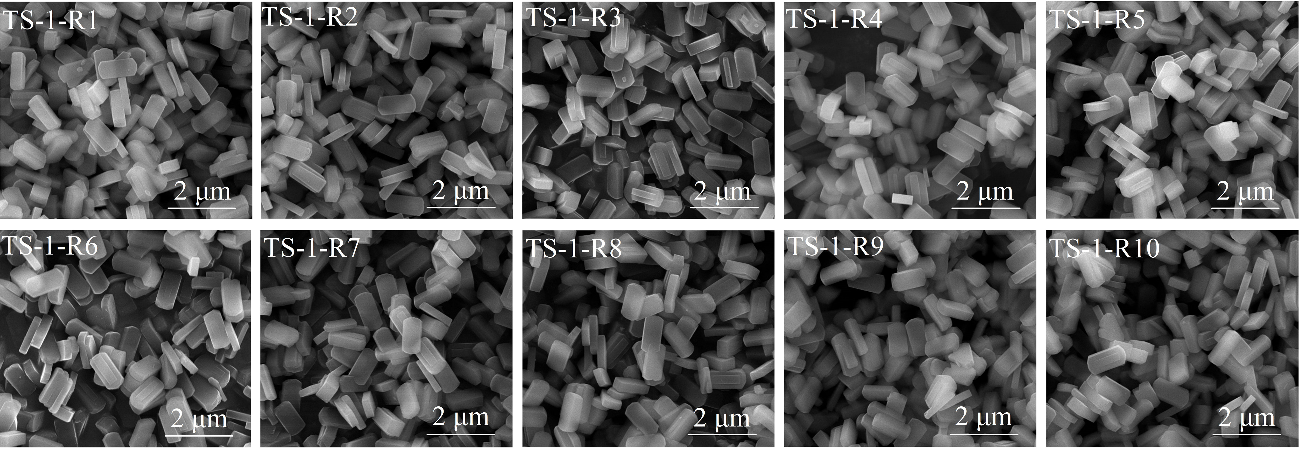


**Supplementary Figure S17.** SEM images of TS-1 samples synthesized with recycled mother liquid from different batches: from 1st batch (TS-1-R1), from 2nd batch (TS-1-R2), from 3rd batch (TS-1-R3), from 4th batch (TS-1-R4), from 5th batch (TS-1-R5), from 6th batch (TS-1-R6), from 7th batch (TS-1-R7), from 8th batch (TS-1-R8), from 9th batch (TS-1-R9), from 10th batch (TS-1-R10).

**
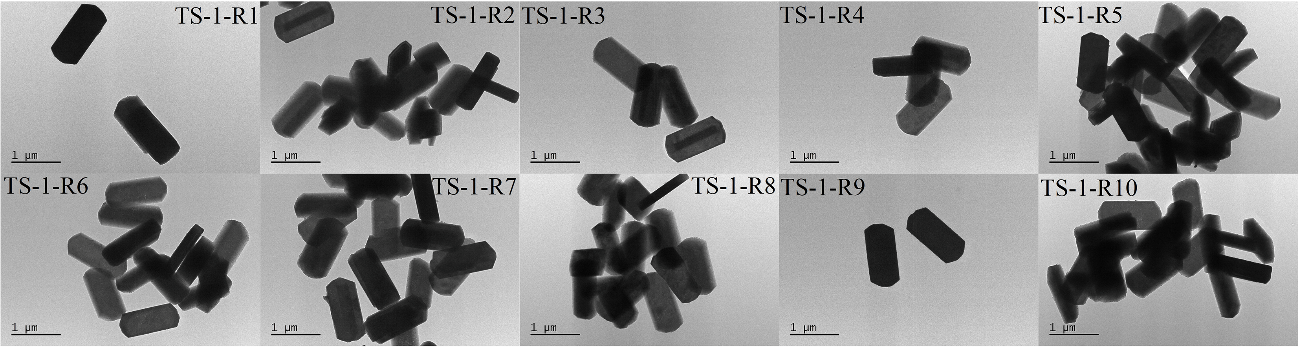
**

**Supplementary Figure S18.** TEM images of TS-1 samples synthesized with recycled mother liquid from different batches: from 1st batch (TS-1-R1), from 2nd batch (TS-1-R2), from 3rd batch (TS-1-R3), from 4th batch (TS-1-R4), from 5th batch (TS-1-R5), from 6th batch (TS-1-R6), from 7th batch (TS-1-R7), from 8th batch (TS-1-R8), from 9th batch (TS-1-R9), from 10th batch (TS-1-R10).


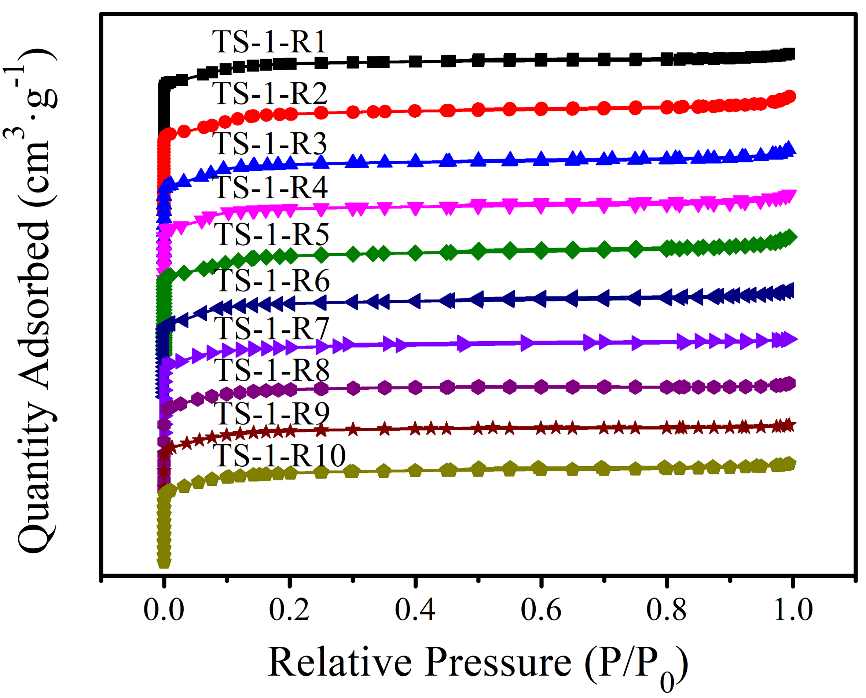


**Supplementary Figure S19.** Nitrogen sorption isotherms of TS-1 samples synthesized with recycled mother liquid from different batches: from 1st batch (TS-1-R1), from 2nd batch (TS-1-R2), from 3rd batch (TS-1-R3), from 4th batch (TS-1-R4), from 5th batch (TS-1-R5), from 6th batch (TS-1-R6), from 7th batch (TS-1-R7), from 8th batch (TS-1-R8), from 9th batch (TS-1-R9), from 10th batch (TS-1-R10).


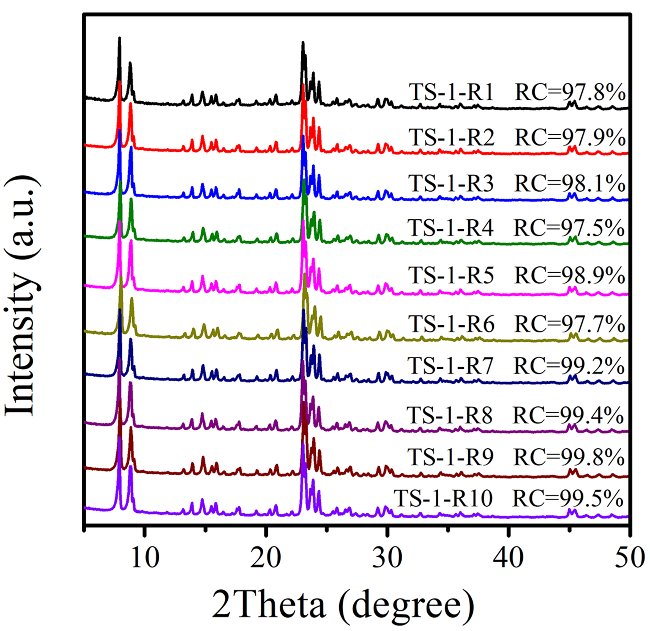


**Supplementary Figure S20.** XRD diffractograms of TS-1 samples synthesized with recycled mother liquid from different batches: from 1st batch (TS-1-R1), from 2nd batch (TS-1-R2), from 3rd batch (TS-1-R3), from 4th batch (TS-1-R4), from 5th batch (TS-1-R5), from 6th batch (TS-1-R6), from 7th batch (TS-1-R7), from 8th batch (TS-1-R8), from 9th batch (TS-1-R9), from 10th batch (TS-1-R10).


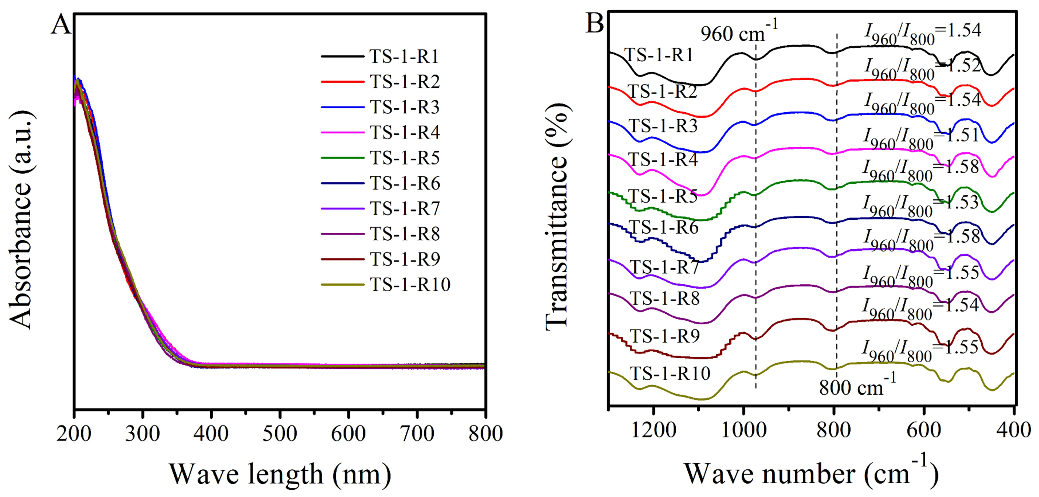


**Supplementary Figure S21.** UV–vis **(A)** and FT-IR **(B)** spectra of TS-1 samples synthesized with recycled mother liquid from different batches: from 1st batch (TS-1-R1), from 2nd batch (TS-1-R2), from 3rd batch (TS-1-R3), from 4th batch (TS-1-R4), from 5th batch (TS-1-R5), from 6th batch (TS-1-R6), from 7th batch (TS-1-R7), from 8th batch (TS-1-R8), from 9th batch (TS-1-R9), from 10th batch (TS-1-R10).

## *Supplementary Tables*

**Supplementary Table S1.** Detailed synthesis conditions of all TS-1 samples.

| Sample | Titanium source | *n*(Si/Ti) | Crystallization time (h) | Ethanolamine amount (mol/L) | TPABr amount (mol/L) | Mother  liquid  recycling |
| --- | --- | --- | --- | --- | --- | --- |
| TS-1-T | TiCl_3_ | 50 | 24 | 0.8 | 0.16 | null |
| TS-1-B | TBOT | 50 | 24 | 0.8 | 0.16 | null |
| TS-1-S | Ti(SO_4_)_2_ | 50 | 24 | 0.8 | 0.16 | null |
| TS-1-30 | TBOT | 30 | 24 | 0.8 | 0.16 | null |
| TS-1-35 | TBOT | 35 | 24 | 0.8 | 0.16 | null |
| TS-1-40 | TBOT | 40 | 24 | 0.8 | 0.16 | null |
| TS-1-45 | TBOT | 45 | 24 | 0.8 | 0.16 | null |
| TS-1-55 | TBOT | 55 | 24 | 0.8 | 0.16 | null |
| TS-1-6h | TBOT | 35 | 6 | 0.8 | 0.16 | null |
| TS-1-12h | TBOT | 35 | 12 | 0.8 | 0.16 | null |
| TS-1-36h | TBOT | 35 | 36 | 0.8 | 0.16 | null |
| TS-1-48h | TBOT | 35 | 48 | 0.8 | 0.16 | null |
| TS-1-60h | TBOT | 35 | 60 | 0.8 | 0.16 | null |
| TS-1-72h | TBOT | 35 | 72 | 0.8 | 0.16 | null |
| TS-1-NEOA | TBOT | 35 | 24 | null | 0.16 | From 1st batch |
| TS-1-0.2EOA | TBOT | 35 | 24 | 0.2 | 0.16 | From 1st batch |
| TS-1-0.4EOA | TBOT | 35 | 24 | 0.4 | 0.16 | From 1st batch |
| TS-1-0.6EOA | TBOT | 35 | 24 | 0.6 | 0.16 | From 1st batch |
| TS-1-0.8EOA | TBOT | 35 | 24 | 0.8 | 0.16 | From 1st batch |
| TS-1-NT | TBOT | 35 | 24 | 0.6 | null | From 1st batch |
| TS-1-0.04T | TBOT | 35 | 24 | 0.6 | 0.04 | From 1st batch |
| TS-1-0.08T | TBOT | 35 | 24 | 0.6 | 0.08 | From 1st batch |
| TS-1-0.12T | TBOT | 35 | 24 | 0.6 | 0.12 | From 1st batch |
| TS-1-R1 | TBOT | 35 | 24 | 0.6 | 0.12 | From 1st batch |
| TS-1-R2 | TBOT | 35 | 24 | 0.6 | 0.12 | From 2nd batch |
| TS-1-R3 | TBOT | 35 | 24 | 0.6 | 0.12 | From 3rd batch |
| TS-1-R4 | TBOT | 35 | 24 | 0.6 | 0.12 | From 4th batch |
| TS-1-R5 | TBOT | 35 | 24 | 0.6 | 0.12 | From 5th batch |
| TS-1-R6 | TBOT | 35 | 24 | 0.6 | 0.12 | From 6th batch |
| TS-1-R7 | TBOT | 35 | 24 | 0.6 | 0.12 | From 7th batch |
| TS-1-R8 | TBOT | 35 | 24 | 0.6 | 0.12 | From 8th batch |
| TS-1-R9 | TBOT | 35 | 24 | 0.6 | 0.12 | From 9th batch |
| TS-1-R10 | TBOT | 35 | 24 | 0.6 | 0.12 | From 10th batch |

**Supplementary Table S2.** The pH values of hydrothermal systems with different titanium sources.

| Ti-source | pH value of hydrothermal system |
| --- | --- |
| TiCl_3_ | 10.56 |
| TBOT | 12.15 |
| Ti(SO_4_)_2_ | 10.23 |

**Supplementary Table S3.** The chemical compositions in the bulk of samples with different titanium sources.*

| Sample | SiO_2_ (wt %) | TiO_2_ (wt %) | *n*_B_(Si/Ti) |
| --- | --- | --- | --- |
| TS-1-T | 97.737 | 2.230 | 58.3 |
| TS-1-B | 97.434 | 2.532 | 51.2 |
| TS-1-S | 97.725 | 2.251 | 57.7 |

**n*_B_(Si/Ti) represents the molar ratio of Si/Ti in the bulk.

**Supplementary Table S4.** Textural properties of samples with different titanium sources.

| Samples | *S*_BET_ | *S*_micro_ | *V*_tot_ | | *V*_micro_ | |
| --- | --- | --- | --- | --- | --- | --- |
|  | m^2^/g | | | cm^3^/g | |  |
| TS-1-S | 417 | 336 | 0.174 | | 0.132 | |
| TS-1-B | 428 | 347 | 0.198 | | 0.142 | |
| TS-1-T | 409 | 328 | 0.165 | | 0.123 | |

**Supplementary Table S5.** Textural properties of samples with different *n*(Si/Ti).

| Samples | *S*_BET_ | *S*_micro_ | *V*_tot_ | | *V*_micro_ | |
| --- | --- | --- | --- | --- | --- | --- |
|  | m^2^/g | | | cm^3^/g | |  |
| TS-1-30 | 425 | 339 | 0.187 | | 0.137 | |
| TS-1-35 | 427 | 341 | 0.195 | | 0.139 | |
| TS-1-40 | 422 | 333 | 0.192 | | 0.135 | |
| TS-1-45 | 429 | 342 | 0.201 | | 0.141 | |
| TS-1-B | 428 | 347 | 0.198 | | 0.142 | |
| TS-1-55 | 426 | 341 | 0.189 | | 0.138 | |

**Supplementary Table S6.** The chemical compositions of obtained solid TS-1 zeolites with different molar ratio of Si/Ti.*

| Sample | SiO_2_ (wt %) | TiO_2_ (wt %) | Si/Ti molar ratio | |
| --- | --- | --- | --- | --- |
|  |  |  | precursor sol  (*n*(Si/Ti)) | solid zeolite  (*n*_B_(Si/Ti)) |
| TS-1-30 | 95.927 | 4.010 | 30 | 31.8 |
| TS-1-35 | 96.532 | 3.451 | 35 | 37.2 |
| TS-1-40 | 96.882 | 3.098 | 40 | 41.6 |
| TS-1-45 | 97.242 | 2.735 | 45 | 47.3 |
| TS-1-B | 97.434 | 2.532 | 50 | 51.2 |
| TS-1-55 | 97.721 | 2.264 | 55 | 57.4 |

**n*_B_(Si/Ti) represents the molar ratio of Si/Ti in the bulk.

**Supplementary Table S7.** The chemical compositions in the bulk of samples with different crystallization time.*

| Sample | SiO_2_ (wt %) | TiO_2_ (wt %) | *n*_B_(Si/Ti) |
| --- | --- | --- | --- |
| TS-1-6 h | 98.132 | 1.859 | 70.2 |
| TS-1-12 h | 97.124 | 2.854 | 45.3 |
| TS-1-35 | 96.532 | 3.451 | 37.2 |
| TS-1-36 h | 96.520 | 3.425 | 37.5 |
| TS-1-48 h | 96.502 | 3.478 | 36.9 |
| TS-1-60 h | 96.532 | 3.454 | 37.2 |
| TS-1-72 h | 96.540 | 3.445 | 37.3 |

**n*_B_(Si/Ti) represents the molar ratio of Si/Ti in the bulk.

**Supplementary Table S8.** Crystal size of TS-1 samples with different crystallization time.

| Sample | Crystallization time (h) | Crystal size (μm) |
| --- | --- | --- |
| TS-1-6 h | 6 | 0.78 × 0.52 × 0.23 |
| TS-1-12 h | 12 | 1.08 × 0.53 × 0.23 |
| TS-1-35 | 24 | 1.08 × 0.52 × 0.24 |
| TS-1-36 h | 36 | 1.09 × 0.51 × 0.25 |
| TS-1-48 h | 48 | 1.07 × 0.51 × 0.25 |
| TS-1-60 h | 60 | 1.09 × 0.52 × 0.24 |
| TS-1-72 h | 72 | 1.09 × 0.53 × 0.24 |

**Supplementary Table S9.** The elemental compositions of samples HTS-1 and NTS-1.*

| Sample | Bulk relative mass  concentration (%) | | | Surface relative atomic  concentration (%) | | |
| --- | --- | --- | --- | --- | --- | --- |
|  | SiO_2_ | TiO_2_ | *n*_B_(Si/Ti) | Si | Ti | *n*_S_(Si/Ti) |
| HTS-1 | 96.266 | 3.612 | 35.4 | 32.4 | 0.16 | 202.5 |
| NTS-1 | 96.475 | 3.490 | 36.8 | 32.1 | 0.42 | 76.4 |

**n*_B_(Si/Ti) and *n*_S_(Si/Ti) represent the molar ratio of Si/Ti in the bulk and on the surface, respectively.

**Supplementary Table S10.** Catalytic activities towards propylene epoxidation of samples HTS-1 and NTS-1.*

| Sample | *X*_H2O2_ (%) | *Y*_PO_ (%) | *S*_PO_ (%) | *U*_H2O2_ (%) |
| --- | --- | --- | --- | --- |
| HTS-1 | 88.5 | 83.0 | 99.1 | 94.6 |
| NTS-1 | 58.6 | 55.6 | 99.3 | 95.5 |

**Reaction conditions*: 318 K; propylene pressure 0.6 MPa; 60 min; cat. 0.03 g; methanol 24 mL; 27.5 wt% H_2_O_2_ 3 mL.

**Supplementary Table S11.** The *TOF* values for samples HTS-1 and NTS-1 and other TS-1 catalysts in literatures.

| Sample | *TOF* (h^-1^) | Ref. |
| --- | --- | --- |
| HTS-1* | 1650 | This work |
| NTS-1* | 1141 | This work |
| TS-1H | 1526 | Our previous work (Liu et al., 2016) |
| ATS-1-0.04 | 696 | (Xiong et al., 2018) |
| HTS-1-5.0 | 168 | (Wang et al., 2019) |
| Sample 6 | 163 | (Zuo et al., 2012) |
| HTS-1 | 110 | (Li et al., 2019) |

**Reaction conditions:* 318 K; propylene pressure 0.6 MPa; 60 min; cat. 0.03 g; methanol 24 mL; 27.5 wt% H_2_O_2_ 3 mL.

*TOF* = *n*_0_(H_2_O_2_) × *X*_H2O2_ × *U*_H2O2_/(*n*_Ti_ × *T*)

The *n*_0_(H_2_O_2_) and *n*_Ti_ represent the initial molar amounts of H_2_O_2_ and Ti of catalyst, respectively. The *X*_H2O2_ and *U*_H2O2_ stand for the conversion of H_2_O_2_ and utilization of H_2_O_2_, respectively. The *T* represents the time on stream.

Li, Y. X., Fan, Q., Li, Y. C., Feng, X., Chai, Y. M., and Liu, C. G. (2019). Seed-assisted synthesis of hierarchical nanosized TS-1 in a low-cost system for propylene epoxidation with H2O2. *Appl. Surf. Sci.* 483**,** 652-660. doi: 10.1016/j.apsusc.2019.03.334.

Liu, M., Chang, Z., Wei, H., Li, B., Wang, X., and Wen, Y. (2016). Low-cost synthesis of size-controlled TS-1 by using suspended seeds: From screening to scale-up. *Appl. Catal. A: Gen*. 525, 59-67. doi: 10.1016/j.apcata.2016.07.006.

Wang, B., Han, H., Ge, B., Ma, J., Zhu, J., and Chen, S. (2019). An efficient hydrophobic modification of TS-1 and its application in the epoxidation of propylene. *New J. Chem.* 43**,** 10390-10397. doi: 10.1039/C9NJ01937E.

Xiong, G., Hu, D., Guo, Z., Meng, Q., and Liu, L. (2018). An efficient Titanium silicalite-1 catalyst for propylene epoxidation synthesized by a combination of aerosol-assisted hydrothermal synthesis and recrystallization. *Microporous Mesoporous Mater.* 268**,** 93-99. doi: 10.1016/j.micromeso.2018.04.015.

Zuo, Y., Wang, M., Song, W., Wang, X., and Guo, X. (2012). Characterization and Catalytic Performance of Deactivated and Regenerated TS-1 Extrudates in a Pilot Plant of Propene Epoxidation. *Ind. Eng. Chem. Res.* 51**,** 10586-10594. doi: 10.1021/ie300581z.

**Supplementary Table S12.** The chemical compositions in the bulk of samples with various EOA contents in the MLRP.*

| Sample | SiO_2_ (wt %) | TiO_2_ (wt %) | *n*_B_(Si/Ti) |
| --- | --- | --- | --- |
| TS-1-NEOA | 99.142 | 0.833 | 158.3 |
| TS-1-0.2EOA | 98.583 | 1.378 | 95.1 |
| TS-1-0.4EOA | 97.925 | 2.061 | 63.2 |
| TS-1-0.6EOA | 96.564 | 3.416 | 37.6 |
| TS-1-0.8EOA | 96.786 | 3.105 | 41.5 |

**n*_B_(Si/Ti) represents the molar ratio of Si/Ti in the bulk.

**Supplementary Table S13.** Textural properties of samples synthesized with various EOA contents in the MLRP.

| Samples | Amount of EOA | *S*_BET_ | *S*_micro_ | *V*_tot_ | | *V*_micro_ | |
| --- | --- | --- | --- | --- | --- | --- | --- |
|  | mol/L | m^2^/g | | | cm^3^/g | |  |
| TS-1-NEOA | 0 | 324 | 228 | 0.307 | | 0.098 | |
| TS-1-0.2EOA | 0.20 | 351 | 268 | 0.268 | | 0.116 | |
| TS-1-0.4EOA | 0.40 | 402 | 306 | 0.190 | | 0.128 | |
| TS-1-0.6EOA | 0.60 | 418 | 335 | 0.185 | | 0.139 | |
| TS-1-0.8EOA | 0.80 | 413 | 329 | 0.182 | | 0.132 | |

**Supplementary Table S14.** The chemical compositions in the bulk of samples with various TPABr contents in the MLRP.*

| Sample | SiO_2_ (wt %) | TiO_2_ (wt %) | *n*_B_(Si/Ti) |
| --- | --- | --- | --- |
| TS-1-NT | 99.653 | 0.106 | 1250.2 |
| TS-1-0.04T | 98.782 | 1.009 | 130.2 |
| TS-1-0.08T | 98.176 | 1.797 | 72.7 |
| TS-1-0.12T | 95.542 | 3.406 | 37.3 |
| TS-1-0.6EOA | 96.564 | 3.416 | 37.6 |

**n*_B_(Si/Ti) represents the molar ratio of Si/Ti in the bulk.

**Supplementary Table S15.** Textural properties of samples synthesized with various TPABr contents in the MLRP.

| Samples | Amount of TPABr | *S*_BET_ | *S*_micro_ | *V*_tot_ | | *V*_micro_ | |
| --- | --- | --- | --- | --- | --- | --- | --- |
|  | mol/L | m^2^/g | | | cm^3^/g | |  |
| TS-1-NT | 0 | 155 | 14 | 0.828 | | 0.008 | |
| TS-1-0.04T | 0.04 | 312 | 220 | 0.386 | | 0.091 | |
| TS-1-0.08T | 0.08 | 405 | 318 | 0.194 | | 0.130 | |
| TS-1-0.12T | 0.12 | 420 | 340 | 0.186 | | 0.140 | |
| TS-1-0.6EOA | 0.16 | 418 | 335 | 0.185 | | 0.139 | |

**Supplementary Table S16.** The chemical compositions in the bulk of samples synthesized by sustainably recycling mother liquid.*

| Sample | SiO_2_ (wt %) | TiO_2_ (wt %) | *n*_B_(Si/Ti) |
| --- | --- | --- | --- |
| TS-1-R1 | 95.542 | 3.406 | 37.3 |
| TS-1-R2 | 96.525 | 3.460 | 37.1 |
| TS-1-R3 | 96.269 | 3.468 | 36.9 |
| TS-1-R4 | 96.581 | 3.398 | 37.8 |
| TS-1-R5 | 96.476 | 3.425 | 37.5 |
| TS-1-R6 | 96.614 | 3.364 | 38.2 |
| TS-1-R7 | 96.489 | 3.407 | 37.7 |
| TS-1-R8 | 96.653 | 3.322 | 38.7 |
| TS-1-R9 | 96.612 | 3.306 | 38.9 |
| TS-1-R10 | 96.589 | 3.330 | 38.6 |

**n*_B_(Si/Ti) represents the molar ratio of Si/Ti in the bulk.

**Supplementary Table S17.** Textural properties of samples synthesized by sustainably recycling mother liquid.

| Samples | *S*_BET_ | *S*_micro_ | *V*_tot_ | | *V*_micro_ | |
| --- | --- | --- | --- | --- | --- | --- |
|  | m^2^/g | | | cm^3^/g | |  |
| TS-1-R1 | 420 | 340 | 0.186 | | 0.140 | |
| TS-1-R2 | 423 | 341 | 0.190 | | 0.144 | |
| TS-1-R3 | 418 | 339 | 0.185 | | 0.135 | |
| TS-1-R4 | 425 | 344 | 0.195 | | 0.145 | |
| TS-1-R5 | 420 | 337 | 0.184 | | 0.137 | |
| TS-1-R6 | 420 | 336 | 0.188 | | 0.136 | |
| TS-1-R7 | 419 | 337 | 0.182 | | 0.138 | |
| TS-1-R8 | 422 | 343 | 0.197 | | 0.144 | |
| TS-1-R9 | 422 | 342 | 0.186 | | 0.143 | |
| TS-1-R10 | 421 | 340 | 0.192 | | 0.142 | |

**Supplementary Table S18.** The yield of zeolite for each batch.

| Sample | Yield of zeolite (%)* |
| --- | --- |
| TS-1-35 | 92.5 |
| TS-1-R1 | 94.3 |
| TS-1-R2 | 94.9 |
| TS-1-R3 | 95.1 |
| TS-1-R4 | 95.4 |
| TS-1-R5 | 96.2 |
| TS-1-R6 | 96.6 |
| TS-1-R7 | 96.5 |
| TS-1-R8 | 97.1 |
| TS-1-R9 | 98.3 |
| TS-1-R10 | 97.9 |

*Yield of zeolite = the mass of obtained solid TS-1 zeolite/the mass of SiO_2_ and TiO_2_ in precursor sol × 100%
